# Supplementary material for: CloneCoordinate: Open-Source Software for Collaborative DNA Construction
Source: ACS Synth Biol. 2025 Nov 18;14(12):4802–18. doi: 10.1021/acssynbio.5c00582 (PMC12723744; doi:10.1021/acssynbio.5c00582)
Supplement: Supplementary file 1 [file sb5c00582_si_001.pdf]

Supplementary Information for

CloneCoordinate: Open-source software for collaborative DNA construction

Ethan Jeon[1], Ziyang Shen[1], Santiago Christ[1], Evelyn Qi[1], Ida Fan[1], Nawon Lee[1], Odysseas Morgan[1], Madeline Ohl[1], Dylan Millson[1], Michelle Laker[1], Aspen Pierson[1], Emily Villegas Garcia[1], Yifan Zhang[1], Adeline Choi[1], Ashrita Iyengar[1], Rebecca Kim[1], Josh Lee[1], Linden Niedeck[1], Vanessa Oeien[1], Maliha Rashid[1], Nandini Seetharaman[1], Arnav Singh[1], Delaney Soble[1], Jenny Yu[1], Katherine Yu[1], Simms Berdy[1], Ellia Chang[1], Robin Kitazono[1], Sofija Ortiz[1], Dylan Taylor[1], and B Thuronyi[1]*

1 Williams College, Williamstown, MA 01267, USA

*To whom correspondence should be addressed. bwt2@williams.edu

Contents

Supplementary Figure 1. Example construct queueing diagram. 2
Supplementary Figure 2. CC Dashboard. 3
Supplementary Figure 3. Flow chart for Assemblies Status code. 4
Supplementary Figure 4. CCA Golden Gate Assembly Queuer tool screenshot. 5
Supplementary Figure 5. CC Experiments tab example. 6
Supplementary Figure 6. Assistant tabs examples. 8
Supplementary Figure 7. CCA Statistics output. 9
Supplementary Figure 8. CCA Operations timeline output. 11
Supplementary Figure 9. CCA Plasmid gel sizing output. 12
Supplementary Figure 10. Additional analyses from CCA Miniprep outcome vs colony count 13
Supplementary Figure 11. Data table for main text Figure 4b. 15
Supplementary Figure 12. Data tables for main text Figure 4c. 16
Supplementary Table 1: Status code flow charts 17
Supplementary Text 1: CloneCoordinate guide and user manual 17
Supplementary Text 2: CloneCoordinate settings documentation for v1.0 17
Supplementary Text 3: CloneCoordinate status documentation for v0.98 24
Supplementary Text 4: Premix documentation for assistant tabs 41

Design — **Constructs 1 and 2 conception & design**

**Tab**	Experiments	
**Sample ID**	e1	
**Type**	control	test
**Draws on**	r1	r2

Queueing phase — **Construct 1 (r1) via Gibson assembly**

**Tab**	Registry	Oligos o				dsDNA d						Assemblies a		
**Sample ID**	r1	o1	o2	o3	o4	d1			d2		d3	a1		
**Type**	construct	primer				PCR			anneal oligos		digest	Gibson		
**Draws on**	external design tools	external design tools				o1	o2	m20	o3	o4	m21	d1	d2	d3

Queueing phase — **Construct 2 (r2) via Golden Gate assembly**

**Tab**	Registry	Golden Gate g				
**Sample ID**	r2	g1				
**Type**	construct	part donor plasmid			PCR	gBlock
**Draws on**	external design tools	r3, m4	r4, m5	r5, m6	r6, d4	r7, o5
	Golden Gate Assembly Queuer					

Correct clone isolation phase — **Construct 1 (r1) straight to minipreps**

**Tab**	Transformations t	*skip optional analytical PCR step*	Minipreps m			Sequencing			Strains
**Sample ID**	t1		m1	m2	m3	serial #			s1
**Type**	chem comp		silica spin column			Sanger	Sanger	Sanger	cryostock
**Draws on**	a1		t1			m1, o5	m2, o5	m2, o6	m2

Correct clone isolation phase — **Construct 2 (r2) analytical PCR on colony stocks**

**Tab**	Transformations t	Analytical PCR	Minipreps m	Sequencing	Strains
**Sample ID**	t2	serial #	m7	serial #	s2
**Type**	electroporation		magnetic bead (SPRI)	nanopore	cryostock
**Draws on**	g1	t2 colony stocks 1-8 o7, o8	t2 colony stock 6	m7	m10

Analysis / quality control steps

**Tab**	dsDNA d	Minipreps m	Analytical PCR
**Type**	Agarose gel electrophoresis		
	UV-vis concentration measurement		

Supplementary Figure 1. Example construct queueing diagram. The diagram shows how two new constructs for a simple experiment might be queued in CC, including example sample identifiers. Both constructs are first designed using existing tools of the user's choice. Construct 1 is queued for a 3-piece Gibson assembly, which includes a PCR (from an existing plasmid template), a pair of annealed oligos, and a restriction digest of an existing plasmid backbone. Construct 2 is queued as a 5-piece Golden Gate assembly which includes 3 existing precloned parts (liberated by type IIS digest of plasmids), an existing PCR product, and a gBlock (ordered as an oligonucleotide and used directly in Golden Gate), each of which is Registered as supplying a Golden Gate part with compatible junctions. The Golden Gate assembly is planned using the Golden Gate Assembly Queuer tool. Next, the constructs are assembled and transformed. Colonies could be miniprepped directly (construct 1) and multiple clones checked by e.g., Sanger sequencing, or colony stocks could be prepared and assessed by analytical PCR (construct 2), then a single clone verified by nanopore sequencing. Verified clones are transformed and stored as strain cryostocks. The Experiment becomes ready to do as soon as both constructs r1 and r2 are sequence-verified.

CloneCoordinate Thuronyi Lab 2019-2025				archived copy								
Recommended (efficient) tasks are highlighted in green and high-priority tasks in lavender + blue. More intense color means the task has not been done in a long time.												
# of priority tasks ready	# of tasks ready	type of sample	procedure	status text shown when ready	# queued	days since last done (25-351)	good # to do	time estimate	protocol quality	task #	type	skill set
0	3	oligos o	make backup and working stocks	ready to stock	2	96	anything ready	1 h	good	1	bench	pipetting, tube strips
0 / 0 / 0	0 / 0 / 0	dsDNA d PCR / anneal / digest	set up PCR / annealing / digest	ready to set up	6	52	8, 16, or 24	1.5 h / 16	good	2	bench	pipetting, tube strips
0 / 0	0 / 0	dsDNA d PCR / digest	purify completed PCRs / digests	ready to clean up		46	up to 24	45 min / 16	fair	3	bench	DNA purification
0	0	dsDNA d	dilute & store annealed oligos	ready to dilute & store		139	anything ready	20 min / 16	good	4	bench	pipetting, tube strips
4	10	assemblies a	assemble from d fragments	ready to assemble	23	63	8, 16, or 24	1.5 h / 16	good	5	bench	pipetting, tube strips
0	0	Golden Gate g	assemble from m donors	ready to assemble	0	56	8	2 h / 16	good	6	bench	pipetting, tube strips
1 / 3	1 / 3	assemblies a / GGate g	transform completed assemblies	ready to transform		27	8, 16, or 24	3 h / 16	good	7	bench	comp cell & Petri dish use
0	0	transformations	score transformation plates	ready to score plates		26	anything ready	2 min each	good	8	bench	colony inspection
0 / 0	0 / 0	assemblies a / GGate g	queue preps of successful transformations/assemblies	ready to queue miniprep			anything ready	10 min	good	9	bench	queueing in CC
15 / 7	18 / 11	minipreps m plates / strains	inoculate liquid culture	ready to inoculate for miniprep	3	26	24 or 48	1h / 24	good	10	bench	inoculation
0	0	minipreps m	prep plasmids after overnight growth (2 day growth max)	ready to miniprep		25	1-2 plates of 24	2 h / 1 plate; 3 h / 2 plates	excellent	11	bench	DNA purification
0 / 20	5 / 21	dsDNA d / minipreps m	quantitate using Take3	ready to quant		62	16, 32, 64...	30 min for 32	good	12	bench	DNA quantitation
0 / 20	3 / 21	dsDNA d / minipreps m	gel sample prep	ready to prep gel sample		57	8, 16, 32...	2 min each	good	13	bench	pipetting, tube strips
0 / 0	0 / 0	transformations / minipreps m	queue analytical PCR	ready to queue analytical PCR		351	as needed	2 min each	fair	14	remote	queueing in CC
0	0	analytical PCR	set up analytical PCR	ready to set up	0	350	8, 16, 32...	1 h for 16	fair	15	bench	pipetting, tube strips
4 / 0	11 / 0	prepped samples / analytical PCRs	load prepped samples, run gel, image gel	ready to run gel		116	>80 samples = full gel	1.5 h	good	16	bench	gel use
0 / 0 / 0	0 / 0 / 0	d / m / analytical PCR	analyze previously run gel	ready to code gel		108	anything ready	15 min per row	poor	17	remote	gel and cloning concepts
0	0	minipreps m (# constructs)	queue sequencing	ready to queue sequencing		35	anything ready	5 min each	good	18	remote	queueing in CC
0 / 0	0 / 0	sequencing Sanger / whole-plasmid	mix sequencing samples	ready to mix	3 / 1	35	anything ready	20 min / 8	good	19	bench	pipetting, tube strips
2	3	sequencing	align sequencing reads to maps	ready to align		32	anything ready	2 min each	fair	20	remote	construct management software
0	5	sequencing	interpret sequencing	ready to interpret		32	anything ready	variable	fair	21	remote	construct management software, cloning
12	147	minipreps m	transform for strain storage	ready to transform for strain storage		134	8 (16 at most)	3 h / 8 to 16	incomplete	22	bench	comp cell & Petri dish use
0	3	minipreps m	inoculate overnight for storage	ready to inoculate for strain storage		133	8, 16, or 24	30 min / 8	fair	23	bench	inoculation
0	0	strains s	store overnight culture as new strain	ready to store		132	8, 16, or 24	30 min / 8	good	24	bench	pipetting, tube strips
	0	experiments e	test and learn from constructs	ready to start	4	293	anything ready	variable	n/a	25	bench	scientific method

0	oligos are ready to order, length ≤ 60
0	oligos are ready to order, length > 60
0	dsDNA or ultramers are ready to order
0	sequencing rxns are queued/mixed for Sanger
0	sequencing rxns are ready to submit for Sanger
0	sequencing rxns are queued/mixed for whole-plasmid sequencing
0	sequencing rxns are ready to submit for whole-plasmid sequencing

CC News 250610 (0 days ago)	• Version 1.0 of CC will shortly be available as a public release! See clonecoordinate.org for details.<br>• News posts for all CC instances will now be pulled from a public Sheet so they can be updated remotely

Supplementary Figure 2. CC Dashboard. The task list reflects the CloneCoordinate Thuronyi Lab 2019-2025 archived CC instance. The task count thresholds for highlighting and the contents of the “good # to do,” “time estimate,” and “protocol quality” columns can be configured in CC Settings. Each row in the “procedure” column can be hyperlinked to protocols, also specified in Settings.

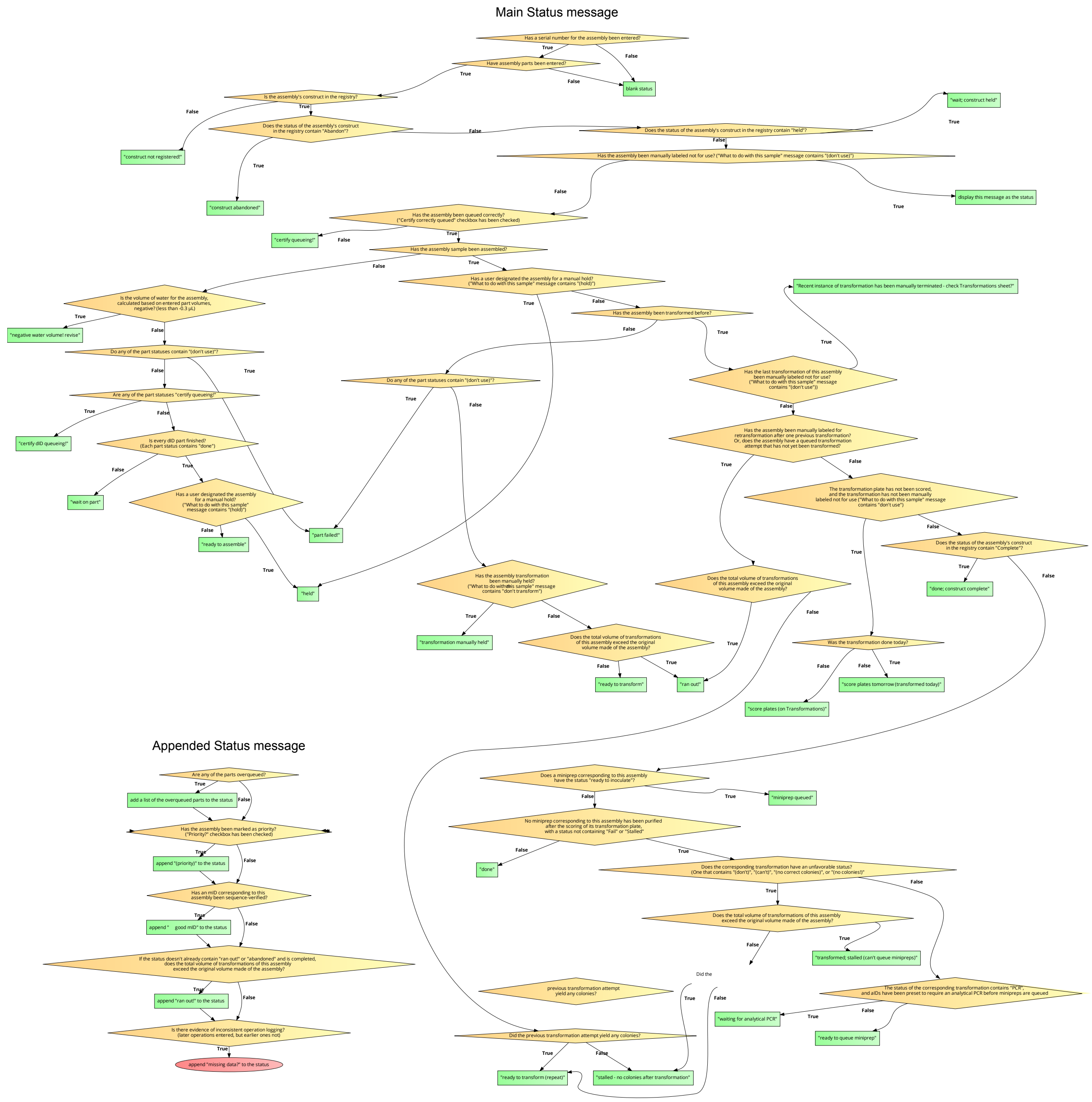

Supplementary Figure 3. Flow chart for Assemblies Status code. Zoom in to view details. The flow chart presents a simplified version of the Sheets formulas that set the Status field for Assemblies. The complete code can be viewed in CC itself. Interactive flow charts for each Status formula are linked from **Supplementary Table 1** and narrative descriptions are in **Supplementary Text 3**.

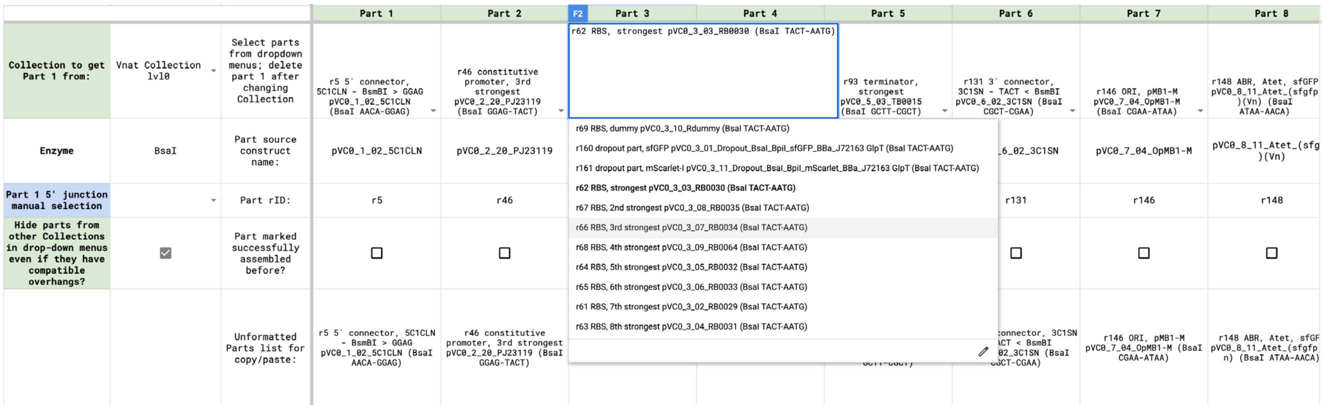

Supplementary Figure 4. CCA Golden Gate Assembly Queuer tool screenshot. An example assembly using parts from the Vnat Collection (Faber, A. *et al. Nucleic Acids Res.* **53**, gkaf580 (2025)) is shown. Working from left to right, parts are chosen from dropdown menus that populate dynamically to display only those parts from the linked CC Registry that will assemble with the previous part, that is, they share the same type IIS enzyme and the 3' junction sequence of the previous part matches the 5' junction sequence of the next part (meaning that the overhang generated at the 3' end of the previous part will be reverse complementary to the overhang generated at the 5' end of the next part). Restriction enzyme, 5' and 3' junction sequences, part descriptive text, sort order, and Golden Gate collection are set in the CC Registry. When connected to a full CC instance (rather than a stand-alone Registry as shown here), the dropdown options will also indicate whether each part is currently in stock (ready to use) based on miniprep inventory. The parts list generated by the tool can be pasted into the Golden Gate g tab of CC to queue an assembly. Palindromic or repeated junctions within the assembly are flagged automatically in the Queuer tool and junction validity is checked when queueing the assembly in CC.

Experiments			1. Sample properties		2a. Queueing, general				
**Serial #**	**ID**	**Status**	**What to do with this experiment?**	**General notes**	**Date queued**	**Queued by**	**Experiment title**	**Short description / experimental question**	**Notebook entry / link**
1	e1	Complete ♥			240719	EJJ	Promoter test	Which promoters have good maximum induction?	https://benchling.com/s/etr-drDfjghaWVObM4kdHcWTj?m=slm-k5sKvz0dsmkr387f
2	e2	Complete ♥			240731	BWT	Strain build	Create new strain	https://benchling.com/s/etr-drDfjghfdjksdgjkhEWdHcWTj?m=slm-kcs,lf38SsmR5uEcZ
3	e3	ready to start			240731	BWT	Burden	Assess metabolic burden for new strain	https://benchling.com/s/etr-drsjdwelkObM4kdHcWTj?m=slm-k5sKvz0dfs394R5uEcZ
4	e4	Required ID is marked ⛔!			240802	ERQ	Output	Measure pathway output for new strain	https://benchling.com/s/etr-drDfjghaWVObcmkfjkeWTj?m=slm-k5sKvfmskfjdk325
5	e5	Wait on constructs			240920	BWT	Homolog test	Replace pathway enzyme with homolog	https://benchling.com/s/etr-drDfjghafdkljEfgeWTj?m=slm-k5sKvfmskfjdk325

					2b. Queueing, constructs/IDs				
**Serial #**	**ID**	**Status**	**Project**	**Assay(s) needed / notes**	**Describe control condition(s)**	**Control construct(s) / IDs needed**	**Describe experimental condition(s)**	**Experimental construct(s) / IDs needed**	**Status needed to consider ID(s) to be ready to use**
1	e1	Complete ♥	Project A	Measure log phase sfGFP expression in plate reader	empty plasmid (no sfGFP); constitutive sfGFP	pBWT001, pBWT002	promoter variants; induce with 1 mM IPTG	pBWT003, pBWT004, pBWT005	
2	e2	Complete ♥	Project B	standard transformation procedure			transform pBWT006 into s25 to create s26	pBWT006, s25	
3	e3	ready to start	Project B	Measure growth curves, OD700 in plate reader	empty strain	s25	measure pathway burden	pBWT006, s26	done
4	e4	Required ID is marked ⛔!	Project B	GCMS on 24h production culture, standard procedure	empty strain, current top producer strain	s25, s6	test new pathway variants	s26, s27, s28	done
5	e5	Wait on constructs	Project C	clone constructs for testing later				pBWT008, pBWT009, pBWT010	

				3. Execution		
**Serial #**	**ID**	**Status**	**Statuses**	**Experiment ready?**	**Notification threshold**	**Email to notify when ready (or status changes)**
1	e1	Complete ♥	pBWT001: Complete ♥<br>pBWT002: Complete ♥<br>pBWT003: Complete ♥<br>pBWT004: Complete ♥<br>pBWT005: Complete ♥	Complete	turn off email notifications for now	ej5@williams.edu, bwt2@williams.edu
2	e2	Complete ♥	pBWT006: Complete ♥<br>s25: OK, done	Complete	notify when any Status changes (may generate lots of emails)	bwt2@williams.edu
3	e3	ready to start	pBWT006: Complete ♥<br>s25: OK, done<br>s26: OK, done	Ready	notify when any Status changes (may generate lots of emails)	bwt2@williams.edu
4	e4	Required ID is marked ⛔!	s26: OK, done<br>s27: Contaminated / heterogeneous! ⛔<br>s28: currently unused (empty)	Required ID is marked ⛔!	notify when experiment is ready or stalled (few emails)	bwt2@williams.edu
5	e5	Wait on constructs	pBWT008: Queued<br>pBWT009: Queued<br>pBWT010: Complete ♥	Wait on constructs		

Supplementary Figure 5. CC Experiments tab example. This tab tracks Experiments which can reference constructs and/or CC sample IDs (such as strains, shown here) as control and/or experimental conditions. It allows CC users to connect their experimental plans to CC's cloning progress so that work can be carried out as soon as the required materials are ready. Users can specify a particular Status that the referenced IDs must have before the Experiment is ready to do (here, strains must be "done," marked complete). Constructs must be completed (cloned) for an Experiment to be ready. If constructs are held or abandoned or any listed samples have problems (indicated by the ⛔ character), the Experiment status

changes to reflect this (e.g., for e4, a required strain was found to be faulty). In addition, the Experiments tab is read into the CCA Construct Tracker Sheet, which maintains information about whether each Queued construct is proceeding through the build process or has become stalled, needing manual intervention to troubleshoot problems. This CCA Sheet has Conditional Notifications which, if enabled, will generate emails to the listed address(es) when constructs become stalled and/or IDs change status according to the selected choices, facilitating prompt attention to problems. The entries are fictitious and for illustrative purposes only.

A

Assist: dsDNA

Plates needed: m05 m06 m07 m37 o10 o12 o13 o15 o16
Premix A: Q5U-MM-1.5x 110.6 µL; Q5U 1.65 µL; 5x CES 31.4 µL; ET-SSB 1.65 µL
Premix B: Q5U-MM-1.5x 73.7 µL; Q5U 1.1 µL; 5x CES 22 µL
Premix C: Q5U-MM-1.5x 73.7 µL; Q5U 1.1 µL; water 22 µL

Sort #	Strip pos'n	ID	Status	Type	Scale (µL)	Queue notes	Premix		Additional component(s)										
1	s1-1	d1365	ready to set up	PCR	50 µL				Q5U-MM-1.5x	33.50 µL	☐	Q5U	0.500	☐	water	7.50	☐	DMSO 2.50	☐
2	s1-2	d1373	ready to set up	PCR	50 µL		Premix A 43.5 µL	☐											
3	s1-3	d1372	ready to set up	PCR	50 µL		Premix A 43.5 µL	☐											
4	s1-4	d1376	ready to set up	PCR	50 µL		Premix B 43.5 µL	☐											
5	s1-5	d1368	ready to set up	PCR	50 µL		Premix B 43.5 µL	☐											
6	s1-6	d1367	ready to set up	PCR	50 µL		Premix C 43.5 µL	☐											
7	s1-7	d1366	ready to set up	PCR	50 µL		Premix C 43.5 µL	☐											
8	s1-8	d1374	ready to set up	PCR	50 µL		Premix A 43.5 µL	☐											

Assist: dsDNA

Blocks needed: 3
63° anneal, 30 cycles (6)
63° anneal, 35 cycles (1)
67° anneal, 35 cycles (1)

Elongation times (minutes:seconds):
63° anneal, 30 cycles - 0:21
63° anneal, 35 cycles - 0:32
67° anneal, 35 cycles - 0:33

Sort #	Strip pos'n	ID	Oligo 1 (well)	µL		Oligo 2 (well)	µL		Input DNA well	Input DNA volume (µL)		# of cycles	Anneal temp (°C)	Required elong (s)	Notes / misc conditions
1	s1-1	d1365	o16.D03	3.00	☐	o16.G03	3.00	☐	m06.D12	0.500	☐	30	63		
2	s1-2	d1373	o16.D03	3.00	☐	o16.E03	3.00	☐	m07.D01	0.500	☐	30	63		
3	s1-3	d1372	o15.A08	3.00	☐	o15.D04	3.00	☐	m05.F07	0.500	☐	30	63		
4	s1-4	d1376	o10.E04	3.00	☐	o12.F09	3.00	☐	m37.B05	0.500	☐	35	63		
5	s1-5	d1368	o15.B11	3.00	☐	o15.G01	3.00	☐	m05.A10	0.500	☐	35	67		
6	s1-6	d1367	o16.F03	3.00	☐	o16.G03	3.00	☐	m06.D12	0.500	☐	30	63		
7	s1-7	d1366	o16.F03	3.00	☐	o16.E03	3.00	☐	m06.D12	0.500	☐	30	63		
8	s1-8	d1374	o13.H04	3.00	☐	o13.A05	3.00	☐	m07.E12	0.500	☐	30	63		

B

Assist: Golden Gate

Premix enabled: see instructions in rows below. Components in premix are grayed out in table.
m plates needed: 19 24 31 36 37 38

Sort #	Strip pos'n	ID	Premix component	µL in premix		µL water		µL Premix		Well, Part 1	µL 1		Well, Part 2	µL 2		Well, Part 3	µL 3		Well, Part 4	µL 4
1	s1-1	g729	*water*	*28.0*	☑	0.146	☐	9.02	☐	*m31.D08*	*0.226*	☑	~~m37.C05~~	~~0.527~~		~~m36.A09~~	~~0.308~~		~~m24.G06~~	~~0.098~~
2	s1-2	g730	*m37.C05*	*2.93*	☑	0.203	☐	9.02	☐	*m31.E08*	*0.226*	☑	~~m37.C05~~	~~0.527~~		~~m36.A09~~	~~0.308~~		~~m24.G06~~	~~0.098~~
3	s1-3	g731	*m36.A09*	*1.711*	☑	0.220	☐	9.02	☐	m36.A08	0.226	☐	~~m37.C05~~	~~0.527~~		~~m36.A09~~	~~0.308~~		~~m24.G06~~	~~0.098~~
4	s1-4	g732	m24.G06	0.544	☐			9.02	☐	m37.C06	0.226	☐	~~m37.C05~~	~~0.527~~		~~m36.A09~~	~~0.308~~		~~m24.G06~~	~~0.098~~
5	s1-5	g733	m37.D05	2.74	☐			9.02	☐	m36.A08	0.226	☐	~~m37.C05~~	~~0.527~~		~~m36.A09~~	~~0.308~~		~~m24.G06~~	~~0.098~~
			m38.F01	2.89	☐															
			m24.H08	3.00	☐															
			NEB 10x T4 ligase buffer	5.55	☐															
			T4 ligase	2.78	☐															

Assist: Golden Gate

Sort #	Strip pos'n	ID	Well, Part 5	µL 5		Well, Part 6	µL 6		Well, Part 7	µL 7		Well, Part 8	µL 8		µL NEB 10x T4 ligase buffer		µL T4 ligase		Enzyme	µL RE	
1	s1-1	g729	~~m37.D05~~	~~0.493~~		~~m38.F01~~	~~0.520~~		~~m24.H08~~	~~0.541~~		m37.E05	0.355	☐	~~1.000~~	☐	~~0.500~~	☐	BsaI	0.250	☐
2	s1-2	g730	~~m37.D05~~	~~0.493~~		~~m38.F01~~	~~0.520~~		~~m24.H08~~	~~0.541~~		m38.H04	0.298	☐	~~1.000~~	☐	~~0.500~~	☐	BsmBI	0.250	☐
3	s1-3	g731	~~m37.D05~~	~~0.493~~		~~m38.F01~~	~~0.520~~		~~m24.H08~~	~~0.541~~		m38.D01	0.281	☐	~~1.000~~	☐	~~0.500~~	☐	BsaI	0.250	☐
4	s1-4	g732	~~m37.D05~~	~~0.493~~		~~m38.F01~~	~~0.520~~		~~m24.H08~~	~~0.541~~		m37.D11	0.501	☐	~~1.000~~	☐	~~0.500~~	☐	BsaI	0.250	☐
5	s1-5	g733	~~m37.D05~~	~~0.493~~		~~m38.F01~~	~~0.520~~		~~m24.H08~~	~~0.541~~		m19.F06	0.405	☐	~~1.000~~	☐	~~0.500~~	☐	BsaI	0.250	☐

Supplementary Figure 6. Assistant tabs examples. The Assistant tabs are populated by user-selected samples from the corresponding tab in order of assigned Sort #. Where applicable, premixes are calculated for samples that are ready for a given task (**Supplementary Text 4**). Check boxes allow users to track which components have been added. **A, Asst: dsDNA showing PCRs. B, Asst: Golden Gate.**

A

Statistics All time	Everyone	B / BWT	Arnav / ADS	Evelyn / ERQ	Katherine / KZY	Maliha / MCR	Santiago / STC	Simms / SB	Sofija / SO
Comprehensive achievement?				🥳🦍🎉					
Oligos o queued	1466	228	4	39	0	0	0	3	0
Oligos o stocked	1461	32	4	129	0	0	0	0	0
dsDNA d queued	1373	299	18	43	16	4	11	0	0
dsDNA d PCRs run	1061	24	23	75	11	0	55	4	0
dsDNA d annealed	292	10	0	28	11	6	1	0	1
dsDNA d purified	1041	70	28	121	25	13	53	4	5
Assemblies a queued	1045	265	0	43	30	3	14	0	0
Assemblies a assembled	957	15	11	102	7	23	21	0	3
Golden Gate g assembled	585	69	0	126	0	13	25	4	0
Transformations	1686	148	27	90	64	54	16	28	16
Plates scored	1634	288	50	56	33	22	22	12	6
Minipreps m queued	3728	955	48	214	16	107	24	0	0
Minipreps m inoculated	3635	417	72	277	24	69	24	24	0
Minipreps m done	3575	386	72	355	0	45	47	24	24
Analytical PCRs run	390	0	0	7	0	0	0	0	0
Sequencing queued	1616	596	0	87	0	3	31	2	0
Sequencing prepared	1600	166	14	101	6	6	32	5	0
Sequencing aligned	1536	698	0	45	23	22	0	0	0
Sequencing interpreted	1486	731	0	46	0	0	0	0	0
Transformations (strain)	200	58	17	16	0	31	0	0	0
Inoculate strain	195	53	25	13	0	49	0	0	0
Strains s stored	795	316	25	19	0	23	0	0	0
dsDNA d / Miniprep m Quantified	4326	185	70	363	18	27	97	12	0
Gel samples prepped	3636	206	92	226	28	14	37	0	3
Gels run	104	9	0	10	5	1	1	1	0
Gel samples coded	3980	584	0	442	61	0	83	0	6
Constructs debugged	235	174	0	14	24	4	15	0	0
Constructs active	778	91	0	50	0	0	1	4	0
Constructs completed	389	43	0	8	0	0	0	2	0
% Constructs complete	50%	47%		16%			0%	50%	
Queued constructs in portfolio	390	51	0	49	0	0	1	2	0
% portfolio complete		77%		17%			0%	50%	

B

Statistics By date range	Show operations that occurred on or after date:		240900	Show operations that occurred on or before date:		250101	
	Everyone	B / BWT	Arnav / ADS	Evelyn / ERQ	Katherine / KZY	Maliha / MCR	Santiago / STC
Oligos o queued	0	0	0	0	0	0	0
Oligos o stocked	33	0	0	0	0	0	0
dsDNA d PCRs run	39	0	3	0	0	0	31
dsDNA d annealed	12	0	0	0	5	6	0
dsDNA d purified	39	4	8	0	0	0	31
Assemblies a assembled	45	0	10	0	0	0	17
Golden Gate g assembled	4	0	0	0	0	0	4
Transformations	76	0	27	1	32	24	0
Plates scored	66	8	50	0	0	0	8
Minipreps m inoculated	96	24	72	0	24	24	0
Minipreps m done	112	40	72	0	0	0	0

C

	Leader, 240900 - 250101
Oligos o queued	
Oligos o stocked	Emily / EVG with 33
dsDNA d PCRs run	Santiago / STC with 31
dsDNA d annealed	Maliha / MCR with 6
dsDNA d purified	Santiago / STC with 31
Assemblies a assembled	Santiago / STC with 17
Golden Gate g assembled	Santiago / STC with 4
Transformations	Katherine / KZY with 32
Plates scored	Arnav / ADS with 50
Minipreps m inoculated	Arnav / ADS with 72
Minipreps m done	Arnav / ADS with 72
Analytical PCRs run	
Sequencing queued	Santiago / STC with 15

Supplementary Figure 7. CCA Statistics output. A, All time task counts for active group members. This view shows the number of tasks (operations on individual samples) completed by group members

who are currently marked active. Jointly completed tasks are credited to all contributors. The highest total for each task is in bold with light gray background. Members such as Evelyn Qi who have completed every task at least once have their contributions shown in lavender and earn celebratory emojis in the top row (“Comprehensive achievement?”). **B, Date range filtered view (abridged)**. Viewers can adjust the start and/or end dates for which to total tasks. **C, Date range leaderboard (abridged)**. This view shows the top contributors and their totals within the selected period. Tasks with no entry were not carried out.

A

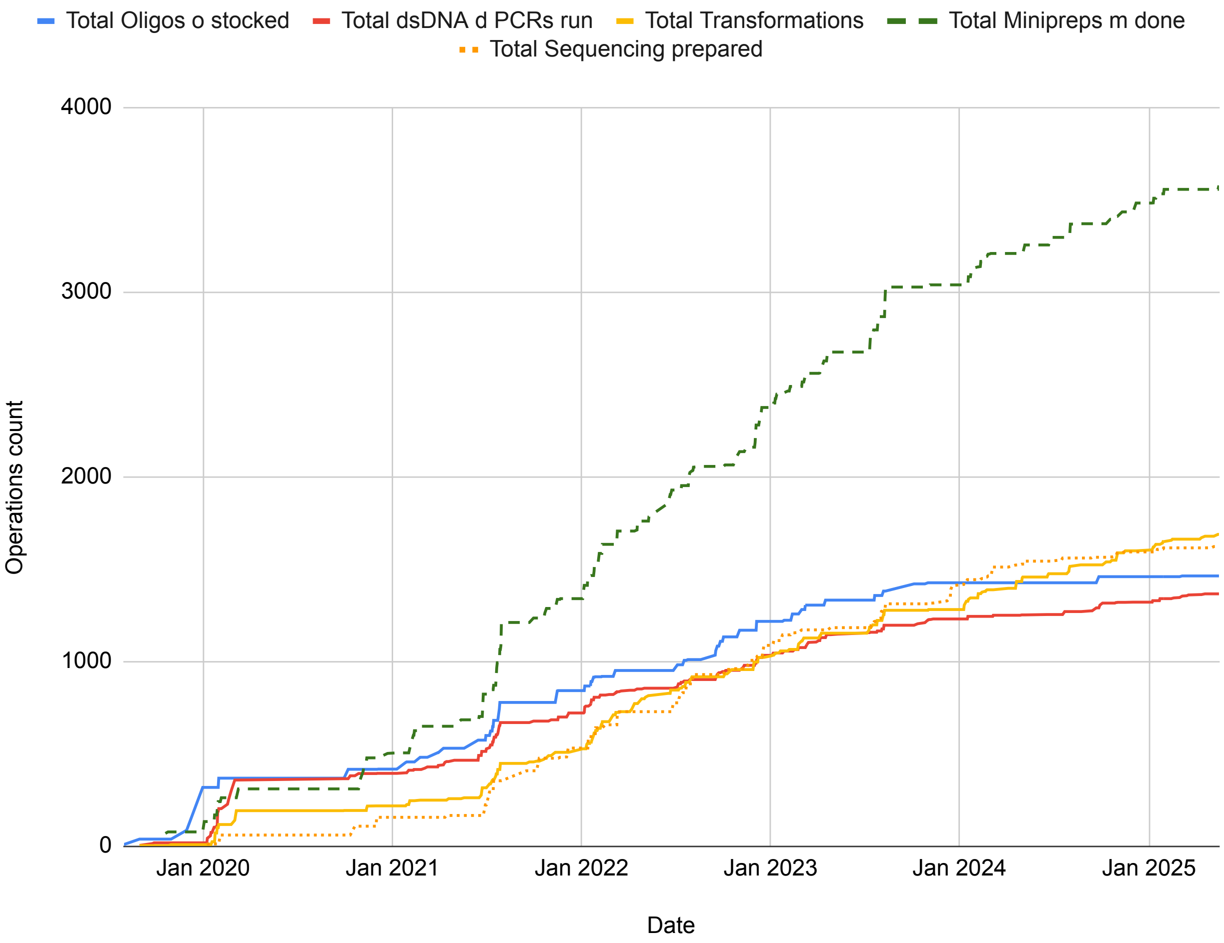

B

Calculate and display this data set on the chart tab?	Operation to tabulate	Cumulative total so far	Average monthly total	Average annual total	Average days per operation
☑	Oligos o stocked	1461	21	250	1
☑	dsDNA d PCRs run	1364	19	234	1.6
☑	Transformations	1686	24	289	1.3
☑	Minipreps m done	3575	51	613	0.6
☑	Sequencing prepared	1620	23	278	1.3

Supplementary Figure 8. CCA Operations timeline output. Up to 5 tasks can be shown at once on this CCA Sheet, including any of those listed on the CC Dashboard as well as construct registration, debugging, and completion. **A, Operations timeline plot.** The cumulative count of selected tasks over time is shown. **B, Operations table.** Totals and monthly, annual, and days per operation (in calendar time, not business days) statistics are tabulated for the selected tasks.

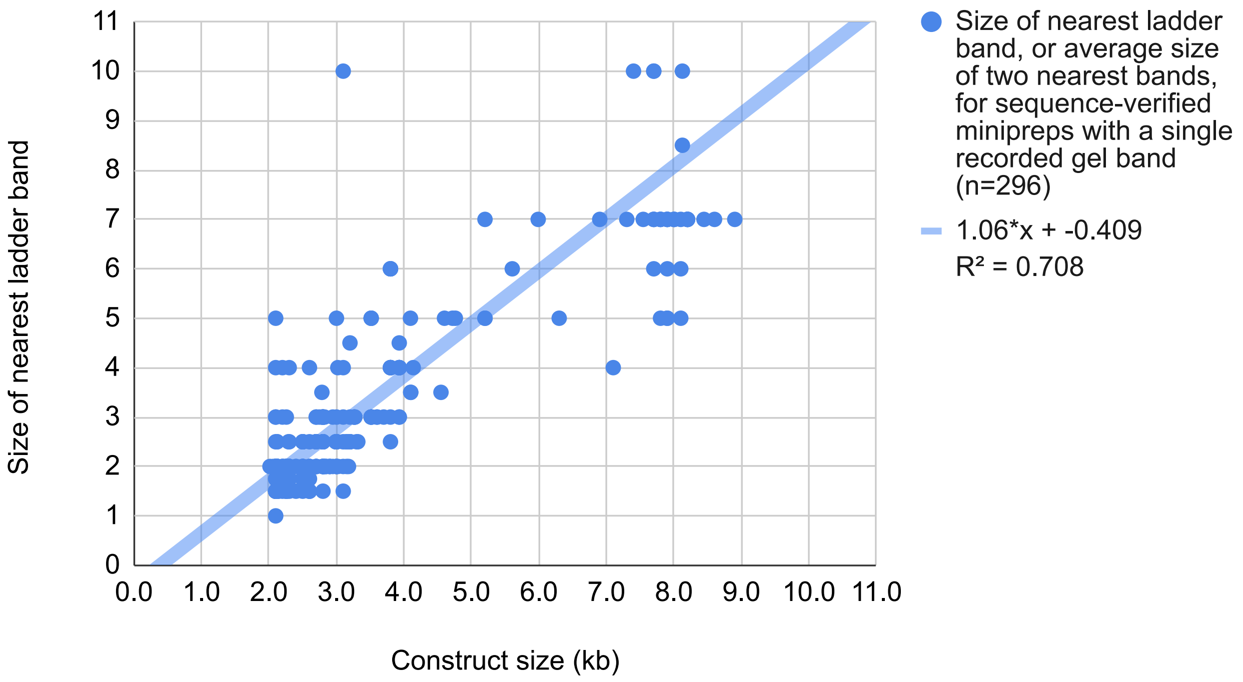

Supplementary Figure 9. CCA Plasmid gel sizing output. The plot shows the size of the nearest band or bands in the sizing ladder selected for agarose gel electrophoresis of sequence-verified minipreps vs. their known size in kilobase pairs. The ladder band size options are quantized. When 2 bands are selected in CC, their average size is used. The fit equation and the square of the Pearson correlation coefficient are shown.

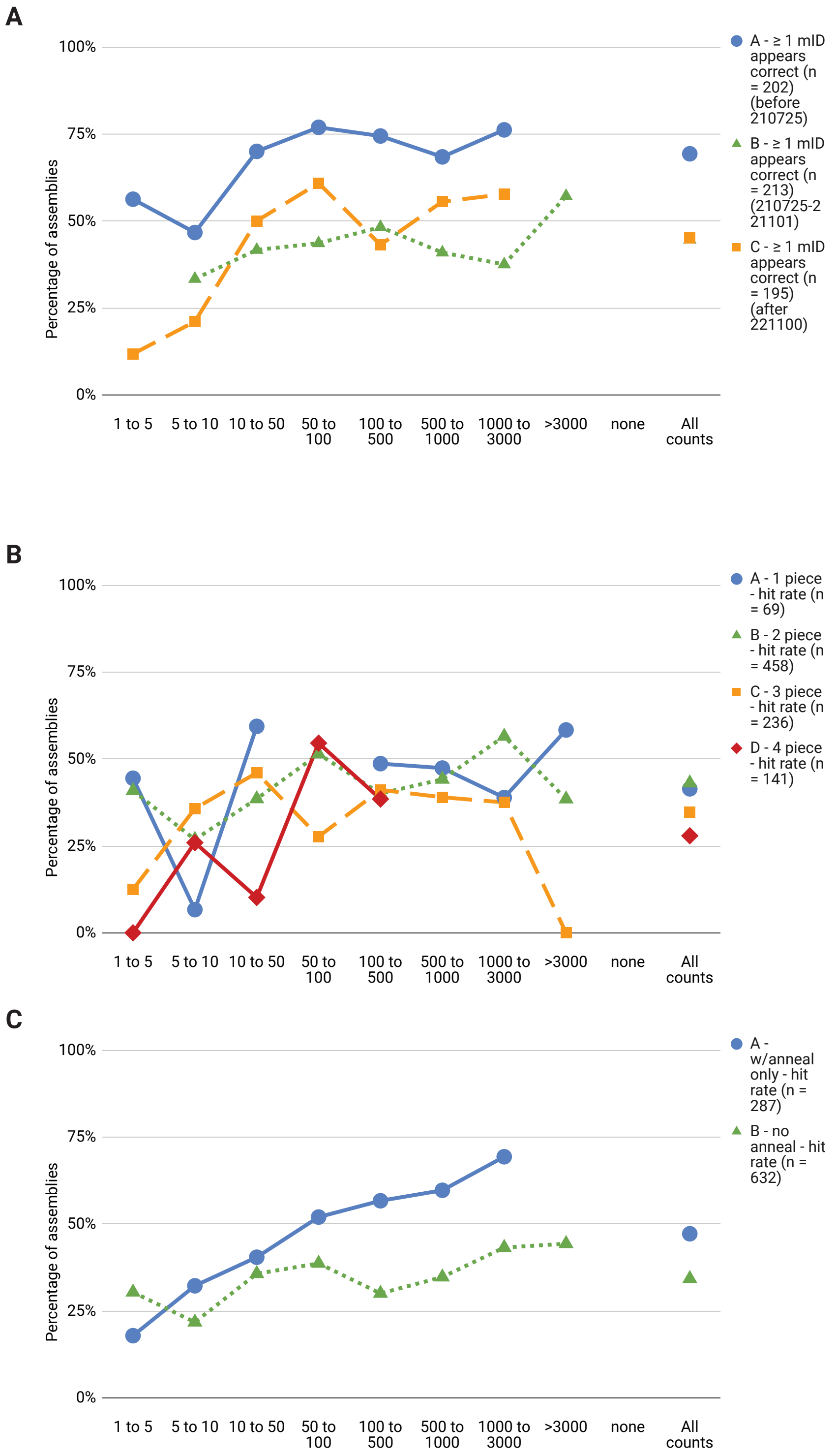

Supplementary Figure 10. Additional analyses from CCA Miniprep outcome vs colony count. The CCA Sheet reports data for USER assemblies (99%) and Gibson assemblies (1%) that resulted in at least one miniprepped colony. **A, Success rate by assembly transformation date**. The plot shows the percentage of assemblies for which at least one clone appears to be correct, based on sequencing and agarose gel electrophoresis data, as a function of colony count (left) or across all assemblies (far right). Percentages based on ≥10 assemblies are shown. The three series are filtered by transformation date

so as to divide assemblies approximately into thirds (transformation before July 25, 2021, blue circles; from July 25, 2021 through November 1, 2022, green triangles; after November 1, 2022, orange squares). The overall success rates are 69%, 45% and 45%. **B, Hit rate by number of parts in assembly.** The plot shows the percentage of assessed clones that appear to be correct for each assembly (the empirical hit rate). Percentages based on ≥5 assemblies are shown. Each series shows assemblies with 1, 2, 3, or 4 separate DNA parts. The overall hit rates are 41%, 43%, 35%, and 28% respectively. **C, Hit rate by presence of annealed oligonucleotide part(s).** The plot shows the empirical hit rate for assemblies that contain (blue circles) or do not contain (green triangles) dsDNA fragments with 3' cohesive end overhangs (generated by annealing partially reverse-complementary oligonucleotides) in addition to PCR products (which are prepared using dU-containing primers, then digested by the USER enzyme mixture to create 3' cohesive end overhangs). The overall hit rates are 47% for assemblies that contain annealed oligonucleotides and 34% for assemblies that do not.

		Colony counts following transformation of assembly									
		1 to 5	5 to 10	10 to 50	50 to 100	100 to 500	500 to 1000	1000 to 3000	>3000	none	**All counts**
**Assembly count by proportion of mIDs appearing correctly assembled based on gel coding**	No mIDs appear correct	20	27	45	34	64	35	28	11		**264**
	Ambiguous or unclear if any mIDs appear correct	3	4	2	3	5	2	3	1		**23**
	At least one mID appears correct	5	4	28	20	41	26	18	10		**152**
	All mIDs appear correct	8	11	29	31	40	24	22	6		**171**
	Total	**36**	**46**	**104**	**88**	**150**	**87**	**71**	**28**		**610**
	% of assemblies, all mIDs appear correct	22%	24%	28%	35%	27%	28%	31%	21%		**28%**
	% of assemblies, ≥ 1 mID appears correct	36%	33%	55%	58%	54%	57%	56%	57%		**53%**
	% of minipreps appearing correct (hit rate)	26%	27%	38%	44%	41%	44%	51%	44%		**40%**
	# minipreps for hit rate calc	50	87	158	129	246	128	91	30		919

Supplementary Figure 11. Data table for main text Figure 4b.

Show assemblies using competent cell batch: 190828-NEB5α		Colony counts following transformation of assembly									
		1 to 5	5 to 10	10 to 50	50 to 100	100 to 500	500 to 1000	1000 to 3000	>3000	**none**	**All counts**
**Assembly count by proportion of mIDs appearing correctly assembled based on gel coding**	No mIDs appear correct	6	3	11	3	11	4	3	1		**42**
	Ambiguous or unclear if any mIDs appear correct	0	2	1	0	0	2	1	0		**6**
	At least one mID appears correct	3	3	11	11	17	21	10	2		**78**
	All mIDs appear correct	7	7	17	13	15	11	8	1		**79**
	Total	**16**	**15**	**40**	**27**	**43**	**38**	**22**	**4**		**205**
	% of assemblies, all mIDs appear correct	44%	47%	43%	48%	35%	29%	36%			**39%**
	% of assemblies, ≥ 1 mID appears correct	63%	67%	70%	89%	74%	84%	82%			**77%**
	% of minipreps appearing correct (hit rate)	48%	66%	46%	72%	53%	68%	68%			**55%**
	# minipreps for hit rate calc	13	10	40	12	51	11	10			147

Show assemblies using competent cell batch: 210720-NEB5α		Colony counts following transformation of assembly									
		1 to 5	5 to 10	10 to 50	50 to 100	100 to 500	500 to 1000	1000 to 3000	>3000	**none**	**All counts**
**Assembly count by proportion of mIDs appearing correctly assembled based on gel coding**	No mIDs appear correct	5	8	16	20	32	14	16	7		**118**
	Ambiguous or unclear if any mIDs appear correct	0	1	1	2	3	0	2	1		**10**
	At least one mID appears correct	2	2	15	10	21	10	6	9		**75**
	All mIDs appear correct	0	3	7	10	12	3	8	3		**46**
	Total	**7**	**14**	**39**	**42**	**68**	**27**	**32**	**20**		**249**
	% of assemblies, all mIDs appear correct	0%	21%	18%	24%	18%	11%	25%	15%		**18%**
	% of assemblies, ≥ 1 mID appears correct	29%	36%	56%	48%	49%	48%	44%	60%		**49%**
	% of minipreps appearing correct (hit rate)	21%	42%	42%	41%	37%	35%	39%	43%		**39%**
	# minipreps for hit rate calc	9	24	58	67	113	51	52	20		394

Show assemblies using competent cell batch: 230223-Mach1		Colony counts following transformation of assembly									
		1 to 5	5 to 10	10 to 50	50 to 100	100 to 500	500 to 1000	1000 to 3000	>3000	**none**	**All counts**
**Assembly count by proportion of mIDs appearing correctly assembled based on gel coding**	No mIDs appear correct	6	11	12	4	9	7	5	1		**55**
	Ambiguous or unclear if any mIDs appear correct	3	1	0	1	2	0	0	0		**7**
	At least one mID appears correct	2	2	6	2	12	5	6	0		**35**
	All mIDs appear correct	1	1	5	6	12	10	6	2		**43**
	Total	**12**	**15**	**23**	**13**	**35**	**22**	**17**	**3**		**140**
	% of assemblies, all mIDs appear correct	8%	7%	22%	46%	34%	45%	35%			**31%**
	% of assemblies, ≥ 1 mID appears correct	25%	20%	48%	62%	69%	68%	71%			**56%**
	% of minipreps appearing correct (hit rate)	34%	13%	40%	48%	58%	63%	62%			**43%**
	# minipreps for hit rate calc	17	35	36	15	33	23	16			175

Show assemblies using competent cell batch: commercial NEB5α C2987U		Colony counts following transformation of assembly									
		1 to 5	5 to 10	10 to 50	50 to 100	100 to 500	500 to 1000	1000 to 3000	>3000	**none**	**All counts**
**Assembly count by proportion of mIDs appearing correctly assembled based on gel coding**	No mIDs appear correct	1	1	1	2	1	0	0	0		**6**
	Ambiguous or unclear if any mIDs appear correct	0	0	0	0	0	0	0	0		**0**
	At least one mID appears correct	0	1	0	2	2	0	0	0		**5**
	All mIDs appear correct	0	0	0	2	1	0	0	0		**3**
	Total	**1**	**2**	**1**	**6**	**4**	**0**	**0**	**0**		**14**
	% of assemblies, all mIDs appear correct				33%						**21%**
	% of assemblies, ≥ 1 mID appears correct				67%						**57%**
	% of minipreps appearing correct (hit rate)				50%						**50%**
	# minipreps for hit rate calc				8						8

Supplementary Figure 12. Data tables for main text Figure 4c.

Supplementary Data

The Thuronyi Lab CloneCoordinate instance (version 0.99) containing cloning data from 2019-2025 can be viewed at the following link. Sequence information and construct descriptions have been removed to maintain privacy for ongoing unpublished research.
https://docs.google.com/spreadsheets/d/1wZwXvLzPqq4h6Fzb-27fKORN6zZ60yFWyrfPe6eCVts

Supplementary Table 1: Status Code Flow Charts

Tab	PDF link
Registry	https://drive.google.com/open?id=1HHxyRm71CwYZZ7UNTTqG4bEiWkNGZKnw
Oligos o	https://drive.google.com/open?id=1V1XlW-vzqCAM5lXqqEnQgyorkOdaVyRo
dsDNA d	https://drive.google.com/open?id=13fSBCXadlrjyDKnvVcWyPMLaw7Fw8-Td
Assemblies a	https://drive.google.com/open?id=1PS1zk2tJW3Qx6e0z-Ss3CORRH54o6hJK
Golden Gate g	https://drive.google.com/open?id=1FjiJxndfZvWR5vl_eVQuABc50wP-LqlZ
Transformations t	https://drive.google.com/open?id=1YHjeDRHrxzzbwaQYCX57Cr2l2Zqu2D0s
Minipreps m	https://drive.google.com/open?id=1IEe4xs23v6Kda5poWpZJXOp7MISjbIN7
Analytical PCR	https://drive.google.com/open?id=14KpKQpCUuIg8m7EJ2-i6xlN5g6KOr8M4
Sequencing	https://drive.google.com/open?id=1w4QFHsSe5HdlqS1GriSp0c_V2eTU7whv
Strains s	https://drive.google.com/open?id=19SEirCOhq9jdFsGvY3GpiP4-GkPV-ZSa

Supplementary Text 1: CloneCoordinate Guide and User Manual

A user manual for CloneCoordinate is available at the following link:
https://docs.google.com/document/d/1Rky-_AYQLumPcQ87oZRaAi_a7sBCuaPWeaZY4-rezh8/

Supplementary Text 2: CloneCoordinate Settings Documentation for v1.0

Settings documentation can be found at the following link, and is reproduced below.
https://docs.google.com/spreadsheets/d/1ifN-zsyOPhla7OHiAEViADX1Y4AJ4UqNp-3klbNFTJM/

Tab(s) affected	Setting name	Description	Named Range designation in CC
**Settings highlighted in purple at the top are the ones you will almost certainly want to adjust when moving into CC for the first time. You can try the default values for the others initially and then change them as needed.**			
Dashboard	Lab name	What is your lab / group's name? (appears on Dashboard)	Settings_DashboardLabName
Dashboard	Protocol links list	Links to lab-specific protocols for each cloning task	Settings_DashboardProtocolLinksList
Registry	Project list	List of projects you are working on	Settings_RegistryProjectList
Assemblies a	Assembly recipes conditions	Components and volumes needed for different assembly recipes that you use	Settings_aAssemblyRecipesConditions
dsDNA d	Cleanup methods	Ways to purify PCR products you use	Settings_dCleanupMethods
dsDNA d	Default cleanup method	Default cleanup method	Settings_dDefaultCleanupMethod
dsDNA d	PCR polymerase components table	Components and volumes needed for each PCR DNA polymerase	Settings_dPCRPolymeraseComponentsTable
dsDNA d	PCR polymerase list	List of DNA polymerases you use for PCRs	Settings_dPCRPolymeraseList
dsDNA d	PCR variation components table	Components and volumes needed for each type of PCR additive	Settings_dPCRVariationComponentsTable
dsDNA d	PCR variation list	List of variations (additives) you use for PCRs	Settings_dPCRVariationList
dsDNA d	Thermocyclers incubators list	List of thermocyclers and/or incubators you use for PCRs and/or DNA assemblies	Settings_dThermocyclersIncubatorsList
Minipreps m	Purification methods	List of methods you use to purify plasmid DNA from bacterial cultures (miniprep)	Settings_mPurificationMethods
Transformations t	Competent cell batches	List of competent cell batches to show in Transformations dropdown menu - add new batches here	Settings_TransformationsCompetentCellBatches
Transformations t	Default comp cell batch	Default comp cell batch (remember to update this when a new batch is made!)	Settings_TransformationsDefaultCompCellBatch
All	Storage plate size	How do you want your physical samples to be stored (other than strains)? This setting affects where people are directed to find samples in the o, d, a, g, and m tabs and several Assistant tabs.<br><br>Enter your choice using the following format:<br>"#w" = # well plate<br>"b#" = box with # spaces<br>"#r#c" = plate with # rows, # cols<br><br>If you're using custom-sized boxes or plates (b# or #r#c) that don't appear in the dropdown menu, you'll need to type in your desired plate/box size using the specified format and ignore the data validation error.	Settings_SampleStoragePlateSize
Analytical PCR	PCR aID require queueing after transformation	For aID transformations: If analytical PCR is called for based on the priority levels set below (aID status shows "ready to queue analytical PCR"), require at least 1 analytical PCR to be queued and completed before the aID is marked "ready to queue miniprep"?	Settings_AnalyticalPCRaIDRequireQueueingAfterTransformation
Analytical PCR	PCR gID require queueing after transformation	For gID transformations: If analytical PCR is called for based on the priority levels set below (gID status shows "ready to queue analytical PCR"), require at least 1 analytical PCR to be queued and completed before the gID is marked "ready to queue miniprep"?	Settings_AnalyticalPCRgIDRequireQueueingAfterTransformation
Analytical PCR	PCR no abandoned constructs	If a construct is marked abandoned in the Registry, prevent analytical PCRs related to that construct from being marked "ready" for any operation?	Settings_AnalyticalPCRNoAbandonedConstructs
Assemblies a	Colony analytical PCR threshold	aID colony analytical PCR threshold	Settings_aIDColonyAnalyticalPCRThreshold
Assemblies a	Default scale	Assembly default scale	Settings_aDefaultScale
Assemblies a	Gibson exact match bases	Gibson number of exactly matching bases required to validate fragment overlap	Settings_aGibsonExactMatchBases
Assemblies a	Gibson premix fraction	Gibson Assembly premix is this fraction of the total assembly volume	Settings_aGibsonPremixFraction
Assemblies a	Gibson search distance	Gibson number of bases at ends of fragments to consider when searching for overlaps	Settings_aGibsonSearchDistance
Assemblies a	Ligation premix fraction	Ligation Assembly premix is this fraction of the total assembly volume	Settings_aLigationPremixFraction
Assemblies a	Miniprep analytical PCR threshold	aID-derived miniprep analytical PCR threshold	Settings_aIDMiniprepAnalyticalPCRThreshold
Assemblies a	Miniprep sequencing require analytical PCR?	For mIDs derived from aIDs: If analytical PCR is called for based on the priority levels set below (mID status shows "ready to queue analytical PCR"), require at least 1 analytical PCR to be queued and completed before the mID is marked "ready to queue sequencing"?	Settings_aIDMiniprepSequencingRequireAnalyticalPCR?
Assemblies a	Overage floor	Overage floor - make at least this much extra premix (μL)	Settings_aOverageFloor

Tab(s) affected	Setting name	Description	Named Range designation in CC
Assemblies a	Overage multiplier	Overage multiplier - call for this fraction of the amount of premix required (unitless)	Settings_aOverageMultiplier
Assemblies a	Premix bias	Premix bias: call for a premix unless NOT premixing saves at least this many pipetting steps. If bias is negative, premix will not be called for even when it saves some steps.	Settings_aPremixBias
Assemblies a	Store in plates?	Store completed assemblies in storage plates (checked) or temporarily as labeled individual samples (unchecked)?	Settings_aStoreInPlates?
Assemblies a	Target nku gibson	Gibson Target molar concentration of each DNA fragment (1 ng/kb/µL for dsDNA = 1.5 nM)	Settings_aTargetNkuGibson
Assemblies a	Target nku ligation	Ligation Target molar concentration of each DNA fragment (1 ng/kb/µL for dsDNA = 1.5 nM)	Settings_aTargetNkµLigation
Assemblies a	Target nku USER	USER Target molar concentration of each DNA fragment (1 ng/kb/µL for dsDNA = 1.5 nM)	Settings_aTargetNkuUSER
Assemblies a	USER premix fraction	USER Assembly premix is this fraction of the total assembly volume	Settings_aUSERPremixFraction
Assemblies a, Golden Gate g	Volume rounding	How do you want to round calculated assembly part volumes, when possible? This affects Assemblies a and Golden Gate g. Not yet implemented for CC v1.0.	Settings_aVolumeRounding
Dashboard	Good number to do list	How many of a task should someone do at once for efficiency (and manageability)	Settings_DashboardGoodNumberToDoList
Dashboard	Highlighting thresholds list	How many samples ready needed for tasks to be highlighted on the Dashboard as efficient choices for work	Settings_DashboardHighlightingThresholdsList
Dashboard	Lab leader name	Lab leader: always displays at the left of stats boards. You can leave this blank if you don't want to set a lab leader.	Settings_DashboardLabLeaderName
Dashboard	Lab member names	Lab member names (used by CCA Statistics)	Settings_DashboardLabMemberNames
Dashboard	Member active?	Is this group member currently working? Used by CCA Statistics.	Settings_DashboardMemberActive?
Dashboard	Protocol quality list	Estimate of how effectively the protocol can lead someone through the task - how much hands-on training should they have first?	Settings_DashboardProtocolQualityList
Dashboard	Remove departed?	Eventually remove departed members from roster?	Settings_DashboardRemoveDeparted?
Dashboard	Time estimate list	How long might it take to do the recommended number of samples for a task?	Settings_DashboardTimeEstimateList
dsDNA d	Agarose gel ladder band sizes	DNA ladder (agarose gels) band sizes in kb; use _1_ to indicate a more intense band at 1 kb	Settings_dAgaroseGelLadderBandSizes
dsDNA d	Agarose gel ladder choices	List of DNA ladders you use, with corresponding entries in ladder band sizes table	Settings_dAgaroseGelLadderChoices
dsDNA d	Anneal buffer volume	Buffer per reaction (µL)	Settings_dAnnealBufferVolume
dsDNA d	Anneal default scale	Standard scale, total reaction volume (µL)	Settings_dAnnealDefaultScale
dsDNA d	Anneal gel quant?	Allow gel sample prep and quantitation for annealed oligo samples?	Settings_QCAnnealGelQuant?
dsDNA d	Anneal min len	Minimum number of bases required for two oligos to be used as annealing inputs (error below this threshold)	Settings_dAnnealMinLen
dsDNA d	Anneal oligo volume	Oligo volume, each oligo (µL)	Settings_dAnnealOligoVolume
dsDNA d	Anneal warn len	Number of bases below which to show a warning for annealed oligos	Settings_dAnnealWarnLen
dsDNA d	Anneal water volume	Water per reaction (µL)	Settings_dAnnealWaterVolume
dsDNA d	Default elution volume	Default elution volume after purification (µL)	Settings_dDefaultElutionVolume
dsDNA d	dID gel sample threshold	dID gel sample threshold	Settings_QCdIDGelSampleThreshold
dsDNA d	dID quant threshold	dID quant threshold	Settings_QCdIDQuantThreshold
dsDNA d	Digest buffer list	Buffer used by each restriction enzyme you work with	Settings_dDigestBufferList
dsDNA d	Digest buffer volumes list	Volume of each buffer needed per standard scale reaction	Settings_dDigestBufferVolumesList
dsDNA d	Digest buffers table	Buffer used by each restriction enzyme you work with	Settings_dDigestBuffersTable
dsDNA d	Digest enzyme list	List of restriction enzymes you use for digests	Settings_dDigestEnzymeList
dsDNA d	Gel dID priority	Designate gel sample prep as a Dashboard task for dIDs when the sample "gel priority" column is set to	Settings_QCGeldIDPriority
dsDNA d	Low digest conc cutoff	Low concentration cutoff for digests - automatically mark PCRs below this value as failed and unusable	Settings_QCLowDigestConcCutoff
dsDNA d	Low digest conc warn	Warning concentration cutoff for digests - flag digests with concentrations below this value as potential failures	Settings_QCLowDigestConcWarn
dsDNA d	Low PCR conc cutoff	Low concentration cutoff for PCRs - automatically mark PCRs below this value as failed and unusable	Settings_QCLowPCRConcCutoff

Tab(s) affected	Setting name	Description	Named Range designation in CC
dsDNA d	Low PCR conc warn	Warning concentration cutoff for PCRs - flag PCRs with concentrations below this value as potential failures	Settings_QCLowPCRConcWarn
dsDNA d	Max samples per block	Maximum number of samples per PCR block	Settings_dMaxSamplesPerBlock
dsDNA d	Min volume to still use	Minimum estimated volume remaining of dID for sheet to validate it as available (µL)	Settings_dMinVolumeToStillUse
dsDNA d	Min volume warn	Volume remaining of dID for sheet to show a warning (µL)	Settings_dMinVolumeWarn
dsDNA d	PCR annealed oligo nku	default nkµ for annealed oligos (multiply by 1.5 for nmolar)	Settings_dPCRAnnealedOligoNku
dsDNA d	PCR assumed conc	Assumed concentration of an unquantitated PCR product (ng/µL)	Settings_dPCRAssumedConc
dsDNA d	PCR default anneal temp	Default anneal temperature	Settings_dPCRDefaultAnnealTemp
dsDNA d	PCR default cycle count	Default cycle count	Settings_dPCRDefaultCycleCount
dsDNA d	PCR default elongation per kb	Default elongation time per kb (seconds)	Settings_dPCRDefaultElongationPerKb
dsDNA d	PCR default polymerase	Default polymerase (from table at right)	Settings_dPCRDefaultPolymerase
dsDNA d	PCR default premix volume per PCR	Premix volume per PCR (everything except oligos and template)	Settings_dPCRDefaultPremixVolumePerPCR
dsDNA d	PCR default scale	Standard scale for PCRs AND digests, total reaction volume	Settings_dPCRDefaultScale
dsDNA d	PCR default variation	Default variation / additive name (from table at right)	Settings_dPCRDefaultVariation
dsDNA d	PCR minimum elongation time	Minimum elongation time (seconds)	Settings_dPCRMinimumElongationTime
dsDNA d	PCR overage floor	Overage floor - make at least this much extra premix	Settings_dPCROverageFloor
dsDNA d	PCR overage multiplier	Overage multiplier - call for this fraction of the amount of premix required (unitless)	Settings_dPCROverageMultiplier
dsDNA d	PCR premix allowed?	Allow Asst: dsDNA to call for premixing reaction components?	Settings_dPCRPremixAllowed?
dsDNA d	PCR premix bias	Premix bias: call for a premix even when premixing requires up to this many extra pipetting steps.	Settings_dPCRPremixBias
dsDNA d	PCR primer volume	Primer volume for this scale, each primer	Settings_dPCRPrimerVolume
dsDNA d	PCR template volume	Template volume - 0 if template volume is neglected	Settings_dPCRTemplateVolume
dsDNA d	Quant dID priority	Designate quantitation as a Dashboard task for dIDs when the sample "quant priority" column is set to	Settings_QCQuantdIDPriority
dsDNA d	Template miniprep min volume left	Minimum estimated volume remaining of mID for sheet to suggest using that mID as a template for PCR	Settings_dTemplateMiniprepMinVolumeLeft
dsDNA d, Minipreps m	Gel sample volume	Upper bound DNA gel sample volume used	Settings_QCGelSampleVolume
Golden Gate g	Default assembly scale	Default assembly scale	Settings_gDefaultAssemblyScale
Golden Gate g	Default buffer vol	Default buffer volume (per default assembly scale)	Settings_gDefaultBufferVol
Golden Gate g	Default ligase vol	Default ligase volume (per default assembly scale)	Settings_gDefaultLigaseVol
Golden Gate g	Default r e vol	Default restriction enzyme volume (per default assembly scale)	Settings_gDefaultREVol
Golden Gate g	Ligase buffer name	What to call the ligase BUFFER used in Golden Gate assemblies? (e.g. specific product name if desired)	Settings_gLigaseBufferName
Golden Gate g	Ligase name	What to call the ligase used in Golden Gate assemblies? (e.g. specific product name if desired)	Settings_gLigaseName
Golden Gate g	Min donor stock conc	Minimum stock concentration required for a miniprep to be designated for use as a Golden Gate donor (nkµ)	Settings_gMinDonorStockConc
Golden Gate g	Min nku GG	Warn in Status when an assembly has a final concentration lower than this for any of its parts (1 ng/kb/µL = 1.5 nM)	Settings_gMinNkuGG
Golden Gate g	Miniprep min conc	Minimum miniprep concentration allowed for sheet to suggest using that mID as a gID component	Settings_gMiniprepMinConc
Golden Gate g	Oligo donor default concentration	Concentration to use for an oligo that is registered as a Golden Gate donor (ng/µL)	Settings_gOligoDonorDefaultConcentration
Golden Gate g	Premix allowed?	Allow Asst: Golden Gate to call for premixing reaction components?	Settings_gPremixAllowed?
Golden Gate g	Premix bias	Premix bias: call for a premix even when premixing requires up to this many extra pipetting steps.	Settings_gPremixBias
Golden Gate g	Premix max enzyme conc factor	What fold concentration can enzymes and buffer be allowed to be in a premix, relative to their 1x final concentrations? (e.g. 2x = twice as concentrated)	Settings_gPremixMaxEnzymeConcFactor
Golden Gate g	Premix overage floor	Overage floor - make at least this much extra premix	Settings_gPremixOverageFloor

Tab(s) affected	Setting name	Description	Named Range designation in CC
Golden Gate g	Store in plates?	Store completed Golden Gate assemblies in storage plates (checked) or as labeled individual samples (unchecked)?	Settings_gStoreInPlates?
Golden Gate g	Target nku golden gate	Default target molar concentration for each part in assemblies	Settings_gTargetNkuGoldenGate
Golden Gate g	Wait time before transforming	Golden Gate assemblies are listed as available to transform this long after the date they are listed as assembled (0 = available immediately)	Settings_gWaitTimeBeforeTransforming
Golden Gate g, Analytical PCR	Colony analytical PCR threshold	gID colony analytical PCR threshold	Settings_gIDColonyAnalyticalPCRThreshold
Golden Gate g, Minipreps m	Gel problem bars use as donor	If a sequence-verified miniprep has agarose gel electrophoresis analysis that suggests a potential problem (e.g. multimerization), prevent that prep from being suggested as a Golden Gate part source?	Settings_gGelProblemBarsUseAsDonor
Golden Gate g, Minipreps m, Analytical PCR	Miniprep analytical PCR threshold	gID-derived mID analytical PCR threshold	Settings_gIDMiniprepAnalyticalPCRThreshold
Golden Gate g, Minipreps m, Analytical PCR	Miniprep sequencing require analytical PCR?	For mIDs derived from gIDs: If analytical PCR is called for based on the priority levels set below (mID status shows "ready to queue analytical PCR"), require at least 1 analytical PCR to be queued and completed before the mID is marked "ready to queue sequencing"?	Settings_gIDMiniprepSequencingRequireAnalyticalPCR?
Minipreps m	Automatic ready to transform option	What newly sequence-verified mIDs should be marked as "ready to transform for strain storage" automatically?	Settings_mAutomaticReadyToTransformOption
Minipreps m	Culture age	If cultures are older than this, discard and grow new cultures for minipreps	Settings_mCultureAge
Minipreps m	Default culture volume	Default culture volume	Settings_mDefaultCultureVolume
Minipreps m	Default media	Default media:	Settings_mDefaultMedia
Minipreps m	Default purification method	Default purification method	Settings_mDefaultPurificationMethod
Minipreps m	Enforce max one prep stored?	Prevent more than one sequence-verified mID from being "ready to transform for strain storage?" even if multiple mIDs are manually set as (store this prep)?<br><br>(If checked, only the first such mID will be set as ready to transform.)	Settings_mEnforceMaxOnePrepStored?
Minipreps m	Gel mID priority	Designate gel sample prep as a Dashboard task for mIDs when the sample "gel priority" column is set to	Settings_QCGelmIDPriority
Minipreps m	Low miniprep conc cutoff	Low concentration cutoff for minipreps- automatically mark mID below this value as failed and unusable	Settings_QCLowMiniprepConcCutoff
Minipreps m	Low miniprep conc warn	Warning concentration cutoff for minipreps - flag mIDs with concentrations below this value as potential failures	Settings_QCLowMiniprepConcWarn
Minipreps m	Media options	List of growth media you use for minipreps	Settings_mMediaOptions
Minipreps m	MID gel sample threshold	mID gel sample threshold	Settings_QCmIDGelSampleThreshold
Minipreps m	MID quant threshold	mID quant threshold	Settings_QCmIDQuantThreshold
Minipreps m	Min volume left	Minimum estimated volume remaining of mID for sheet to suggest using that mID as a gID component	Settings_mMinVolumeLeft
Minipreps m	Plate age	If plates are older than this, show warning in status for "ready to inoculate"	Settings_mPlateAge
Minipreps m	Quant mID priority	Designate quantitation as a Dashboard task for mIDs when the sample "quant priority" column is set to	Settings_QCQuantmIDPriority
Minipreps m	Standard elution volume	Standard elution volume	Settings_mStandardElutionVolume
Minipreps m	Warn volume left	Estimated volume remaining of mID for sheet to warn about using an mID as a gID component	Settings_mWarnVolumeLeft
Minipreps m, Assemblies a	AID analytical PCR after transformation priority	Designate analytical PCR as a Dashboard task for aID-derived transformations when the sample "analytical PCR priority" column is set to	Settings_QCaIDAnalyticalPCRAfterTransformationPriority
Minipreps m, Assemblies a	AID analytical PCR mID priority	Designate analytical PCR as a Dashboard task for aID-derived mIDs when the sample "analytical gel priority" column is set to	Settings_QCaIDAnalyticalPCRmIDPriority
Minipreps m, dsDNA d	Quantitation volume	Upper bound DNA quantitation volume used (for nanodrop, Take3, Qubit, etc.)	Settings_QCQuantitationVolume
Minipreps m, Golden Gate g	GID analytical PCR after transformation priority	Designate analytical PCR as a Dashboard task for gID-derived transformations when the sample "analytical PCR priority" column is set to	Settings_QCgIDAnalyticalPCRAfterTransformationPriority
Minipreps m, Golden Gate g	GID analytical PCR mID priority	Designate analytical PCR as a Dashboard task for gID-derived mIDs when the sample "analytical gel priority" column is set to	Settings_QCgIDAnalyticalPCRmIDPriority
Oligos o	Cost base100nmol plate	Cost per base 100 nmol plate	Settings_oCostBase100nmolPlate

Tab(s) affected	Setting name	Description	Named Range designation in CC
Oligos o	Cost base100nmol tube	Cost per base 100 nmol tube	Settings_oCostBase100nmolTube
Oligos o	Cost base25nmol plate	Cost per base 25 nmol plate	Settings_oCostBase25nmolPlate
Oligos o	Cost base25nmol tube	Cost per base 25 nmol tube	Settings_oCostBase25nmolTube
Oligos o	Cost u100nmol plate	Cost per ideoxyU 100 nmol plate	Settings_oCostU100nmolPlate
Oligos o	Cost u100nmol tube	Cost per ideoxyU 100 nmol tube	Settings_oCostU100nmolTube
Oligos o	Cost u25nmol plate	Cost per ideoxyU 25 nmol plate	Settings_oCostU25nmolPlate
Oligos o	Cost u25nmol tube	Cost per ideoxyU 25 nmol tube	Settings_oCostU25nmolTube
Oligos o	Oligo max length	Length of oligo above which you are not willing to order (bases)	Settings_oOligoMaxLength
Oligos o	Oligo min length	Minimum length of oligos you can / are willing to order (bases)	Settings_oOligoMinLength
Oligos o	Oligo min order	What is the least number of oligos you want to order at once? (e.g. to fulfill the minimum order size for oligos in plates)?	Settings_oOligoMinOrder
Oligos o	Oligo synth scale change len	Length of oligo above which price / synthesis scale changes from your preferred vendor - oligos longer than this will be flagged (bases)	Settings_oOligoSynthScaleChangeLen
Oligos o	Oligo validation substring length diff	When checking for oligos that are substrings of each other, how different in length can the oligos be to consider whether they might be substring matches? (setting this too high will lead to huge numbers of substring matches for long oligos)	Settings_oOligoValidationSubstringLengthDiff
Oligos o	Secondary structure header text	For optional secondary structure entry, what temperature (or other conditions) and/or units should appear in the column header? (Choose whatever preferred design software provides)	Settings_oSecondaryStructureHeaderText
Oligos o	Working concentration	Oligo working solution concentration (for reference only), µM	Settings_oWorkingConcentration
Registry	Antibiotics abbrs	Abbreviations you want to display for the antibiotics you use	Settings_RegistryAntibioticsAbbrs
Registry	Antibiotics descriptions	Names and/or descriptions of the antibiotics you use	Settings_RegistryAntibioticsDescriptions
Registry	Expected min size backbone	Typical or average plasmid backbone size for Golden Gate donor plasmids (everything except the part; kb)	Settings_RegistryExpectedMinSizeBackbone
Registry	Month membership threshold to show	Show only departed members who participated for at least this many months (0 shows all departed members)	Settings_RegistryMonthMembershipThresholdToShow
Registry	Months to keep departed	Months after departure date to keep departed members on the roster for	Settings_RegistryMonthsToKeepDeparted
Registry	Origin compatibility group table	List of replication origin categories (compatibility groups) for plasmids you work with	Settings_RegistryOriginCompatibilityGroupTable
Registry	Sanger read average bases	Estimated average useable bases per read for Sanger sequencing (kbases)	Settings_RegistrySangerReadAverageBases
Registry	Sanger read cost	Sanger sequencing cost per read ($)	Settings_RegistrySangerReadCost
Registry	Warn if not pABC123	Warn if registered construct names don't conform to the pattern pABC123 (except direct receipt constructs)?	Settings_RegistryWarnIfNotpABC123
Registry	Whole plasmid seq cost	Whole plasmid sequencing cost ($)	Settings_RegistryWholePlasmidSeqCost
Sequencing	Primer volume	Primer volume per Sanger sequencing rxn (at default working concentration)	Settings_SequencingPrimerVolume
Sequencing	Require analytical PCR?	If analytical PCRs are queued for an mID, require that they be completed before marking it as ready to queue for sequencing?	Settings_SequencingRequireAnalyticalPCR?
Sequencing	Require gel?	Require gel data coded before marking mIDs as ready to queue for sequencing?	Settings_SequencingRequireGel?
Sequencing	Require QC?	Require QC data before marking dIDs as ready to mix for sequencing?	Settings_SequencingRequireQC?
Sequencing	Require quant?	Require mIDs to be quantitated (concentration determined) before marking them as ready to queue for sequencing?	Settings_SequencingRequireQuant?
Sequencing	Sanger plasmid default conc	Concentration to assume for unquantitated mIDs used in Sanger sequencing (ng/µL)	Settings_SequencingSangerPlasmidDefaultConc
Sequencing	Sanger plasmid targetng	Plasmid ng target per Sanger sequencing reaction	Settings_SequencingSangerPlasmidTargetng
Sequencing	Sanger scale	Sanger sequencing rxn total volume (µL)	Settings_SequencingSangerScale
Sequencing	Whole plasmid conc warning	Whole plasmid sequencing concentration minimum - warn if concentration is below this value:	Settings_SequencingWholePlasmidConcWarning
Sequencing	Whole plasmid high conc threshold	Concentration threshold above which to use "high concentration" volume	Settings_SequencingWholePlasmidHighConcThreshold
Sequencing	Whole plasmid high conc volume	Volume for whole plasmid sequencing for "high concentration" DNA	Settings_SequencingWholePlasmidHighConcVolume
Sequencing	Whole plasmid low conc volume	Volume for whole plasmid sequencing for "low concentration" DNA	Settings_SequencingWholePlasmidLowConcVolume

Tab(s) affected	Setting name	Description	Named Range designation in CC
Strains s	Storage plate size strains	How do you want your physical STRAINS to be stored? This setting affects only the Strains s tab.<br><br>Enter your choice using the following format:<br>"#w" = # well plate<br>"b#" = box with # spaces<br>"#r#c" = plate with # rows, # cols<br><br>If you're using custom-sized boxes or plates (b# or #r#c) that don't appear in the dropdown menu, you'll need to type in your desired plate/box size using the specified format and ignore the data validation error.	Settings_SampleStoragePlateSizeStrains
Transformations t	Default amount	Default volume to transform (µL)	Settings_TransformationsDefaultAmount
Transformations t	Default comp cell vol	Default comp cell volume (to go with default comp cell procedure/batch) (µL)	Settings_TransformationsDefaultCompCellVol
Transformations t	Default growth temp	Default growth temperature (° C)	Settings_TransformationsDefaultGrowthTemp
Transformations t	Default plate amount	Default volume to plate (µL)	Settings_TransformationsDefaultPlateAmount
Transformations t	Default procedure	Default procedure	Settings_TransformationsDefaultProcedure
Transformations t	Default recovery medium	Default recovery medium	Settings_TransformationsDefaultRecoveryMedium
Transformations t	Default recovery time	Default recovery time (min)	Settings_TransformationsDefaultRecoveryTime
Transformations t	Default recovery volume	Default recovery volume (µL)	Settings_TransformationsDefaultRecoveryVolume
Transformations t	Procedures list	List of procedures to show in Transformations dropdown menu	Settings_TransformationsProceduresList

Supplementary Text 3: CloneCoordinate Status Documentation (v0.98)

Portions reproduced with permission from Jeon, E. Developing the CloneCoordinate Suite for Efficient and Informed Synthetic Bacterial Plasmid Construction. Senior Honors Thesis, Williams College (2024).

Registry

The entering of a new serial number indicates the creation of a distinct plasmid construct (an rID), with each construct corresponding to one entire row of the “Registry” spreadsheet. Once the rID has been certified as correctly registered, CloneCoordinate will check for any manual holds or “♥” verifications that have been entered by a user under the “What to do with this construct?” column. Both of these designations will stop any work being done on these constructs - holds, for whatever reason a user may need to place one on a construct, and “♥” verifications, which indicate that the construct’s sequence has been verified as correct. Placing these checks before any other steps ensures that users won’t find themselves doing unnecessary work on any rIDs. However, it also means that some operations may not appear as ready to do for some of the construct’s constituent samples.

Any other manual designations entered under the “What to do with this construct?” column will also appear as the construct status. And any constructs without a properly formatted name - either a user typo or a non-construct entity - will also prompt a warning in their status.

Constructs which are direct receipts are generally treated as sequence-verified. Direct receipts which have an associated strain sample, or sID, but no miniprep, or mID, simply receive the status of "Complete ♥" as they are finished and stored samples. For direct receipts with mIDs, the construct status will tell users to wait until the associated minipreps have been purified and stored.

Of the constructs which are not direct receipts, those with mIDs that have been sequence-verified empirically also receive the "Complete ♥" status. Otherwise, constructs with some associated part (an oligomer oID, double-stranded DNA dID, assembly aID, or Golden Gate gID) will have a “Registered” status. All other constructs will be deemed simply “Queued,” save for those recognized as duplicates of other entered constructs based on their Golden Gate rID composition.

Ranges which Registry Status draws upon:
Minipreps_m_Date_purified, Minipreps_m_Verified_construct?

Oligos o

The entering of a new serial number indicates the creation of a distinct DNA oligomer (Oligo, or oID) sample, with each sample corresponding to one entire row of the "Oligos o" spreadsheet. Clonecoordinate won't return a status unless both a serial number and a nucleotide sequence have been entered for the oID.

If the oID has been certified as correctly queued, CloneCoordinate will then check for any designations containing the symbol "⛔" that have been entered by a user under the "What to do with this sample" column, as well as any manual holds. Placing these checks before any other steps ensures that users won't find themselves doing unnecessary work on any oIDs. However, it also means that no operations whatsoever will be marked as ready to do for samples manually marked ⛔ or (hold).

At this point, the status will advance through the sequential steps of creating the physical oID sample - ordering the oligomer plate, receiving the plate shipment, and making the oligomer stock from the plate (at which point the oID will receive the "done" status).

Certain oID samples themselves can be designated as priority from the Registry by checking the "Flag all related samples as high priority?" column to prioritize all samples related to a construct. This will add them to a separate tally of priority experimental procedures on the CloneCoordinate dashboard and expedite the construction progress of their corresponding constructs. oIDs can also have "missing data?" appended to their status if columns corresponding to later operations have been filled out while earlier ones have not - a sign of inconsistent operation logging.

And finally, CloneCoordinate can also append "not yet used for dsDNA?" - dsDNA meaning double-stranded DNA samples. Excluding oIDs meant to be sequencing or analysis primers, this status tag applies to dsDNA/ultramer oIDs which haven't been entered as a PCR template or as digest DNA for dsDNA, as well as other oIDs not yet entered as PCR primers for dsDNA.

Ranges which Oligos Status draws upon:
dsDNA_d_Input_DNA, dsDNA_d_DNA_to_digest, dsDNA_d_Oligo_1, dsDNA_d_Oligo_2

dsDNA d

The entering of a new serial number indicates the creation of a distinct double-stranded DNA (dsDNA, or dID) sample, with each sample corresponding to one entire row of the “dsDNA d” spreadsheet. dID samples may be one of three types: Anneal (annealed oligos, or oIDs), PCR (amplicons using two oID primers and a template), and Digest (treatment of input DNA, which could be a miniprep or another dsDNA d, with restriction enzymes or some other procedure).

If the dID has been certified as correctly queued, CloneCoordinate will then check for manual holds and any designations containing the symbol "⛔" that have been entered by a user under the “What to do with this sample?” column. Placing these checks before any other steps ensures that users won’t find themselves doing unnecessary work on any dIDs. However, it also means that no operations whatsoever will be marked as ready to do for samples manually marked (hold) or ⛔.

The main branching point in the status’s logic is whether the dID has been mixed and incubated. For those that have not, CloneCoordinate will make sure the component oIDs, if any, are in stock and appropriate for use (the oIDs are not lost, abandoned, or used up, signaled by a "⛔" in their status). At this point, dIDs of the Anneal type will receive the status "ready to set up."

PCR and Digest type dIDs will undergo several more checks of their input DNA (template DNA for PCRs, DNA to be digested for Digests) validity before also proceeding to construction. First, the input DNA needs to be an existing sample in CloneCoordinate - the status will search through every mID, oID, dID, rID, sID, and anything listed within the Registry to find a match. If the template DNA is listed in one of these places, then the template’s sample status is checked. If the input DNA is abandoned, further work on this dID would be pointless. If it is manually held, further work should not be done at this time. In these cases, the dID is marked with the status of the input DNA. If the input DNA’s status does not indicate that it is completed, the dID status will tell users to wait until the input DNA is ready.

dIDs that have been mixed and incubated will receive a status of “done.” These can now be marked as ready for any and all applicable quality control procedures (quantitation and/or gels). Anneals, by default, are not eligible for statuses relating to quantitation/gels unless settings_qcAnnealGelQuant? is changed to TRUE. All eligible samples must also have manual priority designations (in the “Quant priority” and “Gel priority” columns) at least as high as the adjustable thresholds in Settings & admin (at default “low” and “high” priority for quantitation and gels, respectively).

Quality control statuses will be appended to the main status because they are not mandatory to complete plasmid construction in most cases. Therefore, a sample will appear as “done” regardless of the condition of quality control steps, unless a quality control outcome shows the sample is not viable, in which case the “done” status is replaced with “failed”.

CloneCoordinate will flag any dIDs which have been measured to have concentrations too low to be viable for future plasmid construction, based on the appropriate settings_qcLowPCRConcCutoff (currently set at a default 15 ng/µl) or settings_qcLowDigestConcCutoff (default 10 ng/µl). Samples that have not been quantitated or that are above the thresholds are marked as “done”, signaling that they are available and appropriate to use for other operations.

In addition to the minimum concentrations for viability, there are warning thresholds (by default, settings_dMinVolumeToStillUse = 5 µL, settings_qcLowPCRConcWarn = 25 ng/µL, settings_qcLowDigestConcWarn = 20 ng/µL). Viable samples which find themselves below these levels will receive an appended warning status, as a suggestion for users to manually designate them for additional QC checks or be cautious about using them in other operations. A gel result that assesses band position as not matching the dID's expected size will result in a "gel problem?" appendage to the status. This suggests that the PCR amplicon or digest output may not be as intended..

dIDs that go on to be part of an assembly that generates a verified miniprep will also receive the "assembly success ♥" status appendage. This is a mark of some level of trustworthiness since the dID was, at worst, not a complete failure. If given the choice, users might want to use samples of this kind in their assemblies going forward.

Certain dID samples themselves can be designated as priority from the Registry by checking the "Flag all related samples as high priority?" column to prioritize all samples related to a construct. This will add them to a separate tally of priority experimental procedures on the CloneCoordinate dashboard and expedite the construction progress of their corresponding constructs. dIDs can also be deemed to have "no active uses" in their status if they have been added to the Registry list of abandoned dIDs. And dIDs will have "missing data?" appended to their status if columns corresponding to later operations have been filled out while earlier ones have not - a sign of inconsistent operation logging.

Ranges which dsDNA Status draws upon:
settings_qcLowPCRConcCutoff, settings_qcLowDigestConcCutoff, settings_dMinVolumeToStillUse, settings_qcAnnealGelQuant?, settings_QCPriorityList, settings_dMinVolumeWarn, settings_dMinVolumeToStillUse, settings_qcLowPCRConcWarn, settings_qcLowDigestConcWarn

Oligos_o_Status, Oligos_o_ID, Oligos_o_Stocks_made
Minipreps_m_Status, Minipreps_m_ID
dsDNA_d_Status, dsDNA_d_ID
Registry_Status, Registry_ID, Registry_Construct_name, Registry_List_of_abandoned_dIDs
Strains_s_Status, Strains_s_ID

Assemblies a

The entering of a new serial number indicates the creation of a distinct assembly (aID) sample, with each sample corresponding to one entire row of the “Assemblies a” spreadsheet. Clonecoordinate won’t return a status unless both a serial number and at least one assembly part have been entered for the aID.

The status will then run a check on the entered construct for the aID. The construct must also be registered in the “Registry” spreadsheet, and its respective status can’t be abandoned or have a manual hold. The status will check the aID itself for any designations containing the symbol "⛔" that have been entered by a user under the “What to do with this sample” column. Placing these checks before any other steps ensures that users won’t find themselves doing unnecessary work on any aIDs. However, it also means that no operations whatsoever will be marked as ready to do for samples manually marked (hold) or ⛔.

If the aID has been certified as correctly queued, the status will reach the first major branching point in its logic of whether the aID has been assembled. For those that have not, CloneCoordinate will run a series of checks on the calculated assembly conditions and the component parts. The status will alert the user if the entered part volumes for the assembly combined result in a negative volume of water (less than -0.3 μL). The status will also report whether any of the individual part statuses contain "⛔" - signifying some disqualifying issue regarding its part - or indicate incorrect/incomplete queueing or an incomplete part sample. If the assembly does not have a manual hold at this point, its status will mark it as ready to be assembled and list its overqueued parts, if any.

aIDs which have been assembled will first be checked for any manual hold. For those that have not been transformed before, individual part statuses are checked again for “⛔,” as part samples could have been lost or contaminated between the aID’s assembly and its transformation. Then, the status will check for any designations containing the phrase “don’t transform” that have been entered by a user under the “What to do with this sample?” column. This might be an instance where a user wants to simply assemble a plasmid construct without transformation and subsequent testing for verification. If the total volume queued for this transformation doesn’t exceed the original volume made of this aID, the status will mark the aID as ready to transform.

By this point, the remaining aIDs have all had a transformation attempted in the past. If an aID’s most recent transformation attempt was manually terminated (via a "⛔" designation in the Transformations spreadsheet), the aID status will prompt users to look over this last attempt to determine what the best course of action should be before queueing a new transformation attempt for the aID.

For aIDs which have been manually designated for retransformation (from the “What to do with this sample? column in the Assemblies spreadsheet) and those which have new transformation attempts queued but not completed, the status will once again check that the total queued volume for the transformation (including previous attempts) doesn’t exceed the original volume made of the aID. Now these aIDs are ready to be transformed, and statuses will mark transformed plates of aIDs as ready to score as well the day after their transformation, provided that they do not have a "⛔" designation. If a transformation yields no colonies at all, its corresponding aID status will detect that progress on this transformation has been stalled, and further steps such as plate scoring will not appear.

Once a construct has a “complete” status in the registry, its aID will read “done; construct complete” as plate scoring and confirmation of a correct phenotype is the final step of actually assembling a construct - procedures such as sequencing, analytical PCR, and strain storage are technically optional and carried out depending on the constructs’ final purpose.

Some aIDs might be preset to require an analytical PCR before a corresponding mID is queued (Settings_analytical_PCR_ aIDRequireQueueingAfterTransformation, not required by default), and the aID status will make this known to the user. For aIDs which have fulfilled or never had this requirement, the status will indicate that they are ready to have a miniprep queued. Again, the status will check whether any colonies grew from transformation, as well as if the transformation volume exceeded the original aID volume - unsuccessful transformations (those containing “don’t” or “can’t” in their transformation status, or simply grew incorrect colonies) of sufficient remaining aID volume will automatically be labeled for retransformation, while the other unsuccessful transformations will remain stalled and require manual intervention by users for the next steps.

The status will notify the user of any queued minipreps waiting to be inoculated, and if nothing else goes wrong with these minipreps, their aIDs will eventually attain a “done” status.

Certain aID samples themselves can be designated as priority from the Registry by checking the “Flag all related samples as high priority?” column to prioritize all samples related to a construct. This will add them to a separate tally of priority experimental procedures on the CloneCoordinate dashboard and expedite the construction progress of their corresponding constructs. aIDs can also be deemed to have a "♥ good mID" in their status if they have had a corresponding miniprep be empirically sequence-verified. The status will also append whether the aID volume has run out. Even if an aID has run out in this way, users will still be able to view whether it has resulted in a sequence-verified miniprep, as neither of these appended labels are mutually-exclusive. And aIDs will have “missing data?” appended to their status if columns corresponding to later operations have been filled out while earlier ones have not - a sign of inconsistent operation logging.

Ranges which Assemblies Status draws upon:

Settings_analytical_PCR_aIDRequireQueueingAfterTransformation

Registry_Status, Registry_Construct_name
Transformations_Transformed_amount, Transformations_Source_ID,
Transformations_What_to_do_with_this, Transformations_Date_transformed
Minipreps_m_Status, Minipreps_m_Source_ID, Minipreps_m_Date_purified
Lookup_lists_and_tabulations_Verified_mID_Sources

Golden Gate g

The entering of a new serial number indicates the creation of a distinct golden gate (gID) sample, with each sample corresponding to one entire row of the “Golden Gate g” spreadsheet. Clonecoordinate won’t return a status unless a serial number has been entered for the gID.

If the gID has been certified as correctly queued and does not have any designation containing the symbol "⛔" entered by a user under the “What to do with this sample” column, the status will run a check on the entered construct for the gID - if the construct has been abandoned or completed based on its status in the Registry, as well as if it has a manual hold, the gID status will reflect this. Placing these checks before any other steps ensures that no operations whatsoever will be marked as ready to do for these samples so that users won’t find themselves doing unnecessary work on any gIDs.

Now, the status will reach the first major branching point in its logic of whether the gID has been assembled. For those that have not, CloneCoordinate will run a series of checks on the calculated assembly conditions and the component parts. The status will alert the user if the entered part volumes for the assembly combined result in a negative volume of water (less than -0.3 µL). The status will also report on the state of the constructs from which the individual golden gate parts are derived. CloneCoordinate will detect whether any parts fail to be sourced to constructs, whether they have yet to be cloned, whether they need to be stocked and have their concentrations determined, and whether any have received a manual hold.

At this point, if the golden gate itself does not have a manual hold, the status will prompt the user to enter the part mIDs and volumes for the assembly. Any invalid mID inputs (where the mID does not correspond with the rID) will be pointed out, as well as whether the part junctions remain valid with these mIDs. Otherwise, the status will indicate that the golden gate is ready for assembly. If any part concentrations are lower than the preset threshold (settings_gMinNkuGG, by default 0.3 ng/kb/µL), CloneCoordinate will append a warning to the “ready to assemble” status. A list of overqueued parts, if any, will also be appended.

If there is no manual hold, the status will now advance to deal with gIDs which have been assembled. Users can preset the amount of time which must pass before assembled gIDs can be queued for transformation (settings_gWaitTimeBeforeTransforming, by default one day) as well as enter a “don’t transform” designation under the “What to do with this sample” column, both of which will affect whether the gID will appear as “ready to transform.” The status will check for and rule out transformations of gIDs for which more total volume has been queued for transformation than was made of the gID itself. And if a gID’s most recent transformation attempt was manually terminated (via a "⛔" designation in the Transformations spreadsheet), the gID status will prompt users to look over this last attempt to determine what the best course of action should be before queueing a new transformation attempt for the gID. Otherwise, gIDs will be marked as ready to transform.

For gIDs which have been manually designated for retransformation (from the “What to do with this sample? column in the Golden Gate spreadsheet) and those which have new transformation attempts queued but not completed, the status will once again check that the total queued volume for the transformation (including previous attempts) doesn’t exceed the original volume made of the gID. Now these gIDs are ready to be transformed, and statuses will mark transformed plates of gIDs as ready to score as well the day after their transformation, provided that they do not have a "⛔" designation. If a

transformation yields no colonies at all, its corresponding gID status will detect that progress on this transformation has been stalled, and further steps such as plate scoring will not appear.

Once a construct has a “complete” status in the registry, its gID will read “done; construct complete” as plate scoring and confirmation of a correct phenotype is the final step of actually assembling a construct - procedures such as sequencing, analytical PCR, and strain storage are technically optional and carried out depending on the constructs’ final purpose.

The status will check for unsuccessful transformations (those containing “don’t” or “can’t” in their transformation status, or simply grew incorrect colonies or none at all) will be labeled as stalled and require manual intervention by users for the next steps. Some gIDs might be preset to require an analytical PCR before a corresponding mID is queued (Settings_analytical_PCR_ gIDRequireQueueingAfterTransformation, not required by default), and the gID status will make this known to the user. For gIDs which have fulfilled or never had this requirement, the status will indicate that they are ready to have a miniprep queued or, if queued, waiting to be inoculated. If nothing else goes wrong with these minipreps, their gIDs will eventually attain a “done” status.

Certain gID samples themselves can be designated as priority from the Registry by checking the “Flag all related samples as high priority?” column to prioritize all samples related to a construct. This will add them to a separate tally of priority experimental procedures on the CloneCoordinate dashboard and expedite the construction progress of their corresponding constructs. gIDs can also be deemed to have a "♥ good mID" in their status if they have had a corresponding miniprep be empirically sequence-verified. The status will also append whether the gID volume has run out. Even if an gID has run out in this way, users will still be able to view whether it has resulted in a sequence-verified miniprep, as neither of these appended labels are mutually-exclusive. And gIDs will have “missing data?” appended to their status if columns corresponding to later operations have been filled out while earlier ones have not - a sign of inconsistent operation logging.

Ranges which Golden Gate Status draws upon:

settings_gMinNkuGG, settings_gWaitTimeBeforeTransforming,
Settings_analytical_PCR_gIDRequireQueueingAfterTransformation

Registry_Status, Registry_Construct_name, Registry_GG_parts_display_name, Registry_5_overhang, Registry_ID, Registry_3_overhang
Minipreps_m_Construct_name, Minipreps_m_ID
Transformations_Transformed_amount, Transformations_Source_ID,
Transformations_What_to_do_with_this, Transformations_Date_transformed
Minipreps_m_Status, Minipreps_m_Source_ID, Minipreps_m_Date_purified
Lookup_lists_and_tabulations_Verified_mID_Sources

Transformations

The entering of a new serial number indicates the creation of a distinct transformation attempt, with each transformation corresponding to one entire row of the “Transformations” spreadsheet. If the transformation has been certified as correctly queued, CloneCoordinate will then check that the corresponding construct of the source ID exists in the Registry spreadsheet, as well as whether this construct has been abandoned or placed on a manual hold by a user under the “What to do with this sample?” column in the Registry. Placing these checks before any other steps ensures that users won’t find themselves doing unnecessary work on any transformations for constructs of jeopardized status.

Transformations themselves, as well as their source IDs (aIDs, gIDs, and mIDs) can be given designations containing the symbol "⛔" by users under the “What to do with this sample?” column in their respective spreadsheets. All of these will similarly halt or terminate progress on their transformations. If no such designations are present, then we are ready to transform. The status will make note of whether this is a first-time transformation or a repeat, and whether previous transformation attempts have been carried out or simply queued.

The status will warn the user if the combined volume of queued transformation attempts for a source ID fall short of the preset default transformation volume (settings_transformationsDefaultAmount, set to 5 μL by default) - transformation attempts using extremely small volumes can result in suboptimal colony growth. The status will also warn again if any parts have received a "⛔" designation for transformations of aIDs. Both of these warnings are appended to the appropriate “ready to transform” status, as the transformation may still be carried out, just with suboptimal results.

Plates are to be scored a day after the transformations are done, and any plates with no colonies grown will be treated as a finished transformation attempt (a status of “done,” appended with “no colonies!”). Plates with colony growth but no correct phenotypes will also finish with an appendage noting their ineligibility for accurate miniprep queueing.

This status can also call for analytical PCR to be run following transformation. Source aIDs and gIDs each have their own preset priority threshold level for warranting an analytical PCR - settings_aIDColonyAnalyticalPCRThreshold and settings_gIDColonyAnalyticalPCRThreshold, respectively, each comprised of four levels (“do not do,” “low,” “normal,” and “high”) and set to “high” by default (mIDs, by default, are set between “do not do” and “low,” allowing analytical PCRs in general to be run but not qualifying them for any thresholds requiring at least a “low” priority). If a user manually enters an analytical PCR priority for a transformation which matches or exceeds these preset thresholds, that transformation will receive a status of "ready to queue analytical PCR.” The status will reflect ongoing progress on analytical PCRs until their completion, upon which their transformations will receive a “done” status.

Certain transformations themselves can be designated as priority from the Registry by checking the “Flag all related samples as high priority?” column to prioritize all samples related to a construct. This will add them to a separate tally of priority experimental procedures on the CloneCoordinate dashboard and expedite the construction progress of their corresponding constructs. Transformations will also have “missing data?” appended to their status if columns corresponding to later operations have been filled out while earlier ones have not - a sign of inconsistent operation logging.

Ranges which Transformations Status draws upon:

settings_transformationsDefaultAmount, settings_aIDColonyAnalyticalPCRThreshold, settings_gIDColonyAnalyticalPCRThreshold, settings_qcPriorityList

Registry_Status, Registry_Construct_name
Assemblies_a_What_to_do_with_this, Assemblies_a_ID
Golden_Gate_g_What_to_do_with_this, Golden_Gate_g_ID
Minipreps_m_What_to_do_with_this, Minipreps_m_ID
Transformations_Source_ID, Transformations_Date_transformed, Transformations_Transformed_amount

Minipreps m

The entering of a new serial number indicates the creation of a distinct miniprep (mID) sample, with each sample corresponding to one entire row of the “Minipreps m” spreadsheet. Clonecoordinate will return a blank status until a serial number has been entered for the mID, then will prompt the user until the mID has been certified as correctly queued.

Minipreps are a very central sample type within CloneCoordinate - they act as a link between aIDs and gIDs, their transformations (tIDs), and many quality control procedures. The mID status will first check for any discrepancies between the manual status designations entered for the mID (under the “What to do with this sample?” columns in both the Minipreps and Sequencing spreadsheets), then for any manual holds entered in either spreadsheet (specifying from which it comes from). Conflicting manual designations may be an indication of incorrect information at some point in CloneCoordinate, or they may be misinputs themselves. If quantitated, a miniprep with a concentration below the preset cutoff minimum (15 ng/μL, by default) will be given a failed status. Minipreps with corresponding constructs that have been abandoned or manually held will similarly be held, and those with no growth following inoculation will also be labeled as stalled. Placing these checks before any other steps ensures that users won’t find themselves doing unnecessary or incorrect procedures on any mIDs.

The status will label mIDs as stalled if the culture antibiotic does not match that of the construct marker - this will mean the growth from the culture may contain other bacteria than that of the desired plasmid. Then following a successful inoculation, the status will prompt users to prepare a miniprep, given that a day has passed since inoculation but not enough days to exceed the preset maximum time (Settings_mCultureAge, by default two days) to disqualify a culture as too old to miniprep.

Any manual status designation on a transformation (under the “What to do with this sample?” column in Transformations) will disqualify its associated mID from further steps until removed. CloneCoordinate also has a “Plate status” column which performs its own checks on the transformation plates for each mID - for mIDs derived from aIDs and gIDs, the mID status will call for a retransformation if no available transformation plate is detected, and it will also prompt users to manually examine the transformation plate if its status has deemed it to be of questionable age. mIDs with positive plate statuses will be labeled as ready for inoculation. For mIDs derived from sIDs at this stage, the mID status will wait until the corresponding strains have been stored before labeling the mID as ready for inoculation.

For any remaining mIDs which have not been purified, those with no source ID entered will return a blank status, and all others will appear as “stalled.”

As long as their volumes have not run below the preset minimum (Settings_mMinVolumeLeft, 5.00 μL by default), purified minipreps will receive a “done” status. Any manual hold (from either Minipreps or Sequencing) will be appended, specifying from which spreadsheet it comes from. The status will also append whether the miniprep growth was an unexpected phenotype/color.

There are many paths that a miniprep could take from this point, not necessarily in any order, so these minipreps will receive many status appendages for the different procedures they can receive. mIDs with manual priority designations for DNA quantitation and gel electrophoresis (in the “Quant priority” and “Gel priority” columns) at least as high as the adjustable thresholds 'Settings & admin'!$BH$29 and 'Settings & admin'!$BH$27 (both set to “normal” priority by default) will appear ready for these steps in their

statuses. Minipreps of aIDs and gIDs for which users have entered analytical PCR priority at least as high as their preset thresholds (settings_aIDMiniprepAnalyticalPCRThreshold and settings_gIDMiniprepAnalyticalPCRThreshold, both set to “high” priority by default) will also appear ready for analytical PCR. The mID status will also advance through the stages of a gel - prepping the sample, running the gel, then coding the gel - as they are completed.

The mID status will also append whether a miniprep should be sequenced. This depends on a number of checks. The construct corresponding to the mID must have been checked off for sequencing by a user (under the “Currently needs sequencing?” column in the Registry). If quality control procedures (quantitation, gel, analytical PCR) have been preset as required before sequencing (via settings_SequencingRequireQuant?, settings_SequencingRequireGel?, and settings_SequencingRequireAnalyticalPCR?, all required by default), they must also be completed before “ready to queue sequencing” is appended to the mID status. The analytical PCR requirement for sequencing can also further be specified between mIDS derived from aIDs and gIDs (via settings_aIDMiniprepSequencingRequireAnalyticalPCR? and settings_gIDMiniprepSequencingRequireAnalyticalPCR?, both disabled by default as they are overridden by settings_SequencingRequireAnalyticalPCR?). There is also a final “Is sequencing appropriate?” column in Minipreps which rules out additional scenarios which would disqualify mIDs from sequencing (those with rID and sID sources, those from colonies of unexpected phenotype/color, etc.) - any result in this column containing “Do not” will disqualify mIDs from sequencing, while a result of “Check QC” will direct users to manually review the ambiguous quality control data and decide whether to manually queue the mIDs for sequencing.

The mID status will append warnings for mIDs which fall below the preset warning threshold, but above the preset working minimum, for volume (Settings_mWarnVolumeLeft and Settings_mMinVolumeLeft), as well as those below the preset warning threshold for concentration (settings_qcLowMiniprepConcWarn). CloneCoordinate also has a system for rating the quality and intensity of gel results as good, problematic, or ambiguous (via the pairs of settings_GelCodingQualityFlags with settings_GelCodingQuality and settings_GelCodingIntensityFlags with settings_GelCodingIntensity) - if any problematic results are detected, a warning will be appended to the mID status of this possibility.

The setting settings_mAutomaticReadyToTransformOption allows users to preset which newly sequence-verified mIDs should be marked as "ready to transform for strain storage" automatically - all of them, only new ones manually designated for storage, or none. While currently set to automatically mark all by default, the status will check for manual designations should the setting be adjusted. And those which have been transformed in this way will then be marked as ready to be inoculated for storage.

Certain mID samples themselves can be designated as priority from the Registry by checking the “Flag all related samples as high priority?” column to prioritize all samples related to a construct. This will add them to a separate tally of priority experimental procedures on the CloneCoordinate dashboard and expedite the construction progress of their corresponding constructs.

mIDs can also receive the "♥ verified" status appendage if they have been sequence-verified. This applies to mIDs derived from sIDs which have been designated to reprep their respective plasmids and do not have any "⛔" manual designation (under the “What to do with this sample?” columns in the

Minipreps, Sequencing, or Strains spreadsheets). All mIDs of rIDs will automatically appear as verified. mIDs of any remaining source ID types (such as aIDs and gIDs) will appear as verified if they have the verification indicator "♥" in a manual designation (under the "What to do with this sample?" columns in the Minipreps or Sequencing, specified if from Sequencing), with an appended note if the compiled sequencing data does not definitely indicate sequence verification (in the "Compiled sequencing results" column).

And mIDs will have "missing data?" appended to their status if columns corresponding to later operations have been filled out while earlier ones have not - a sign of inconsistent operation logging.

Ranges which Minipreps Status draws upon:

settings_qcLowMiniprepConcCutoff, Settings_mMinVolumeLeft,
settings_aIDMiniprepAnalyticalPCRThreshold, settings_gIDMiniprepAnalyticalPCRThreshold
settings_qcPriorityList, settings_SequencingRequireQuant?, settings_SequencingRequireGel?
settings_SequencingRequireAnalyticalPCR?, settings_gIDMiniprepSequencingRequireAnalyticalPCR?,
settings_aIDMiniprepSequencingRequireAnalyticalPCR?, Settings_mWarnVolumeLeft,
settings_qcLowMiniprepConcWarn, settings_GelCodingQualityFlags, settings_GelCodingQuality,
settings_GelCodingIntensityFlags, settings_GelCodingIntensity,
settings_mAutomaticReadyToTransformOption, Settings_mCultureAge

Registry_Manual_status_assignment, Registry_Construct_name, Registry_Needs_sequencing?
Strains_s_Status, Strains_s_Storage_strain?, Strains_s_Date_stored
Transformations_What_to_do_with_this, Transformations_Source_ID

Analytical PCR

The entering of a new serial number indicates the creation of a distinct analytical PCR sample, with each sample corresponding to one entire row of the “Analytical PCR” spreadsheet. If the analytical PCR has been certified as correctly queued, CloneCoordinate will then check for manual holds and any designations containing the phrase “don’t use” that have been entered by a user under the “What to do with this sample?” column. Placing these checks before any other steps ensures that users won’t find themselves doing unnecessary work on any analytical PCRs. However, it also means that no operations whatsoever will be marked as ready to do for samples manually marked (hold) or ⛔.

CloneCoordinate will also check the status of the construct corresponding to the analytical PCR. Abandoned constructs can be preset to never have analytical PCRs performed on their samples (via Settings_analytical_PCR_NoAbandonedConstructs, true by default), and constructs with manual holds will result in holds for their analytical PCRs as well.

Before an analytical PCR is run, its status will check through the statuses of its PCR components. Both the forward and reverse oligomer oIDs must be finished and without any designations containing the phrase “don’t use” entered by a user under the “What to do with this sample?” column on the “Oligos o” spreadsheet. The template DNA sample - aID, gID, mID, or sID - must also be finished. The analytical PCR can be run at this point, and afterward the status will proceed through the sequential steps of running, and then coding, a corresponding gel sample.

Analytical PCRs will have “missing data?” appended to their status if columns corresponding to later operations have been filled out while earlier ones have not - a sign of inconsistent operation logging.

Ranges which Analytical PCR Status draws upon:
Settings_analytical_PCR_NoAbandonedConstructs
Registry_Status, Registry_Construct_name
Oligos_o_Status, Oligos_o_ID

Sequencing

The entering of a serial number indicates the creation of a distinct sequencing sample, with each corresponding to one entire row of the “Sequencing” spreadsheet. Source IDs can be either dIDs or mIDs, and sequencing can be either Sanger or whole-plasmid.

If the sequencing has been certified as correctly queued, CloneCoordinate will then check for any designations containing the symbol "⛔" that have been entered by a user under the “What to do with this sample?” column, as well as manual holds. Placing these checks before any other steps ensures that users won’t find themselves doing unnecessary work on any sequencing. However, it also means that no operations whatsoever will be marked as ready to do for samples manually marked ⛔ or (hold).

The main branching point in the status’s logic is whether the sequencing sample has been mixed. For those that have not, CloneCoordinate will make sure its constituent source ID and sequencing primer oID samples don’t have some disqualifying status containing “⛔,” and that these samples have been finished. By default, CloneCoordinate also requires quality control data on dIDs before marking them as ready to mix for sequencing - this setting, settings_SequencingRequireQC?, can be toggled off as needed. oIDs satisfying all of these checks are ready to mix.

CloneCoordinate can now append a series of warnings to the status as applicable, starting with a possible redundancy warning for sequencing samples for completed, abandoned, or manually held template constructs. Whole-plasmid sequencing samples will also receive a warning if their template concentration falls below the preset warning threshold settings_SequencingWholePlasmidConcWarning (30ng/µl by default).

The appended warning checks for source ID volume are more extensive. The status will warn users if the current remaining volume of any source dID falls below the preset warning threshold settings_dMinVolumeWarn (10µl by default). And based on how much of the source ID has been queued in various samples across all of CloneCoordinate, the status will tell users whether the source ID volume is going to fall below the preset working minimums - settings_dMinVolumeToStillUse and Settings_mMinVolumeLeft for dIDs and mIDs, respectively, both 5µl by default - or below the warning threshold from above.

Of the sequencing samples that have been mixed, those that have been manually omitted from sequencing under the “What to do with this sample?” column will be stamped with a status of “done”, so that no further steps will unnecessarily appear in their status. The remaining sequencing samples are then sequentially guided through the steps of assigning a well to the mixed sample, submitting the sample and receiving the sequencing read, and aligning and interpreting the read. Some alignments may be classified as not usable or not required to be interpreted, and these will be given a “done” status as well. The remaining alignments are then classified into “(easy)” and “(hard)” interpretations based on whether they completely match their expected nucleotide sequence.

Certain sequencing samples themselves can be designated as priority from the Registry by checking the “Flag all related samples as high priority?” column to prioritize all samples related to a construct. This will add them to a separate tally of priority experimental procedures on the CloneCoordinate dashboard and expedite the construction progress of their corresponding constructs. Sequencing samples can also have

“missing data?” appended to their status if columns corresponding to later operations have been filled out while earlier ones have not - a sign of inconsistent operation logging.

Ranges which Sequencing Status draws upon:
settings_SequencingRequireQC?, settings_SequencingWholePlasmidConcWarning, settings_dMinVolumeWarn, settings_dMinVolumeToStillUse, Settings_mMinVolumeLeft

Registry_Status, Registry_Construct_name
Oligos_o_Status, Oligos_o_ID
dsDNA_d_Status, dsDNA_d_Volume_left
Minipreps_m_Status, Minipreps_m_Volume_left

Strains s

The entering of a new serial number indicates the creation of a distinct strain storage sample, with each sample corresponding to one entire row of the “Strains s” spreadsheet. Unlike most other statuses, the strains status will first check whether this strain - corresponding to a position on a well or other physical storage setup - is unused and empty. Prominently noting empty wells is important because strains are in cryostorage. In terms of the spreadsheet, this would mean that the user has entered nothing under the columns for transformed DNA, plasmid name, organism, strain, and date stored.

If the strain has been certified as correctly queued, CloneCoordinate will then check for manual holds and any designations containing the symbol "⛔" that have been entered by a user under the “What to do with this sample?” column. Placing these checks before any other steps ensures that users won’t find themselves doing unnecessary work on any strains. However, it also means that no operations whatsoever will be marked as ready to do for samples manually marked (hold) or ⛔.

The status will update as the DNA to be transformed is inoculated, then as this miniprep is successfully stored as a strain.

The user can select “Empirically verified correct ♥” under the “What to do with this sample?” column to append “♥ verified” to the status, an indication that the sequence of this stored strain has been verified through sequencing.

Certain strain storage samples can be designated as priority from the Registry by checking the “Flag all related samples as high priority?” column to prioritize all samples related to a construct. This will add them to a separate tally of priority experimental procedures on the CloneCoordinate dashboard and expedite the construction progress of their corresponding constructs. And strains will have “missing data?” appended to their status if columns corresponding to later operations have been filled out while earlier ones have not - a sign of inconsistent operation logging.

Ranges which Strains Status draws upon:
Minipreps_m_Date_inoculated_for_storage

Supplementary Text 4: Premix Documentation for Assistant Tabs

Several *in vitro* assembly tasks, as well as media preparation for culture inoculation, can benefit from premixing some of the components shared in common between multiple samples. CloneCoordinate has code to generate labor-saving (though not necessarily optimal) premixes for Asst: dsDNA, Asst: Assemblies, Asst: Golden Gate, and Asst: Minipreps. The Golden Gate premix code provides a good overview of the approach used. Premixes for other Assistant tabs are calculated along similar lines.

Golden Gate assemblies combine multiple plasmid and/or PCR product parts, known as Golden Gate donors, and assemble them scarlessly into a desired construct using a type IIS restriction enzyme and T4 DNA ligase. CloneCoordinate's Golden Gate assembly assistant sheet offers users the option to enhance efficiency and reduce pipetting errors by employing a premix during the assembly process. This premix contains standardized reaction components common to all assemblies being conducted. Importantly, the current premixing algorithm will only call for premixing components that have identical volumes in every currently selected assembly. To use a premix, the user must choose, by manually assigning sort numbers, only assemblies that share common components and ensure that they need identical volumes. Additional assemblies with a separate set of common components can then be carried out separately afterward. Implementation of a more robust and automatic premix selection algorithm is a future development goal.

Within the Golden Gate section of the settings/admin sheet, users can specify whether to activate the assistant's recommendation for using a premix (default = premixing enabled). If users opt against using a premix, the assistant will not enable premixing formulas. Otherwise, the assistant suggests premixing when the number of pipetting operations required without premixing exceeds those with a premix. The decision determines the practicality of premix utilization by comparing the sum of premixed components and assembly counts against the product of these two values. The former sum corresponds to the number of pipetting operations to (1) add each component to the premix and then (2) pipette premix into each reaction; the latter is the number of operations to put each component into each reaction separately. Users also have the option to input a custom premix bias in settings (Settings_gPremixBias, default value = 1, premixing recommended even if it requires 1 additional operation), recommending premixing even when it marginally increases pipetting operations. This flexibility acknowledges that the slight increase in pipetting may be outweighed by the improved accuracy and/or consistency that premixing allows. If premixing is calculated to be efficient based on these criteria, the assistant sheet systematically identifies components shared across assemblies and designates these as premix components. Enzymes and buffers are included in the premix provided their concentrations do not exceed a maximum fold concentration relative to their 1x final concentrations, a parameter inputted by the user in settings (Settings_gPremixMaxEnzymeConcFactor, default value = 2.0x). Moreover, the premix calculation accounts for the total number of assemblies utilizing the premix and determines the

volume required, with an adjustable added overage volume configurable in the settings (Settings_gPremixOverageFloor default value = 5.00 µL) to allow for residual liquid adhering to tubes and tips during work. The total volume of premix dispensed into each assembly tube is the sum of individual component volumes within the premix. By streamlining the assembly process and minimizing human error through strategic premixing, CloneCoordinate's Golden Gate assembly assistant sheet enhances efficiency while accommodating user-specific preferences and settings.

The technical implementation of the premix calculations is as follows:

For each row, components ("Well, Part 1," "Well, Part 2," etc…) and their respective volumes ("µL 1," "µL 2," etc…) are concatenated in a list. Within this list, components and their accompanying volumes are delineated by a comma (,) while different components are separated by vertical bars (|), all stored in the "Component-volume list for this row" cell. Additionally, the type IIS restriction enzyme ("µL RE"), T4 DNA ligase ("µL T4 ligase"), and T4 DNA ligase buffer ("µL T4 ligase buffer") for each assembly are appended to the end of this cell in the same format.

In a new column, "Component sources to potentially premix, " the assistant sheet parses through each "Component-volume list for this row" cell, separating the list at each vertical bar (|). It then compares the formatted component and volume pairs across all the rows. If multiple assemblies use identical components with matching volumes, these shared components are listed in the "Component sources to potentially premix" column, with their respective volumes in the "Volumes to potentially premix" column.

To maintain enzyme and buffer concentrations within acceptable limits relative to their 1x final concentrations when added to the premix solution, a validation check is used. In the "Component sources to premix" column, components identified in the "Component sources to potentially premix" column are included unless they are enzymes or buffers. Enzymes and buffers are conditionally added based on user-defined maximum concentration parameters configured in settings (settings_gPremixMaxEnzymeConcFactor).

To determine the amount of each component to add into the premix, the volume of each component required for an individual assembly (as listed in the "Volumes to premix" column) is multiplied by the number of assemblies requiring that particular component. This product is further multiplied by an overage factor, determined by a custom setting ("settings_gPremixOverageFloor") within the Golden Gate section of the settings/admin sheet. Finally, the total premix volume ("µL Premix") for each assembly is calculated as the sum of volumes listed in the "Volumes to premix" column.

The Asst: dsDNA and Asst: Assemblies premix code operates largely analogously to the Golden Gate premix code, with separate configuration Settings. These premixes are simpler and so the decision about whether to create a premix is carried out separately for each sample, and the premix is called for if it reduces pipetting operations (accounting for the premix bias Setting). These Assistant tabs can recommend multiple premixes for a single set of samples. The Asst: Minipreps tab simply applies an overage factor to each antibiotic media type needed, and recommends a premix of media and antibiotic

even for small numbers of wells/tubes needing the media type, since these are simple to account for manually.
